# Total Fat and Fatty Acid Intake among 1–7-Year-Old Children from 33 Countries: Comparison with International Recommendations

**DOI:** 10.3390/nu13103547

**Published:** 2021-10-09

**Authors:** Cathriona Monnard, Mathilde Fleith

**Affiliations:** Nestlé Institute of Health Sciences, Nestlé Research, Société des Produits Nestlé S.A., 1015 Lausanne, Switzerland; mathilde.fleith@gmail.com

**Keywords:** fat, fat intake, nutrients, nutrient intake, children, DHA, EPA

## Abstract

This work reviews available data on dietary intakes of total fat, saturated fatty acids (SFA) and individual polyunsaturated fatty acids (PUFA) in children in different countries worldwide and for the first time, compares them with recent international recommendations. Studies published before June 2021 reporting total fat, total SFA and individual PUFA intakes in children aged 1–7 y were included. Observed intakes were evaluated against FAO/WHO and EFSA recommendations. 65 studies from 33 countries were included. Fat intake was too low in 88% of studies in young children (1–3 y). SFA intake was >10%E in 69–73% of children, especially in Europe. Linoleic acid intake was <3%E in 24% of studies in 1–2 y olds and within FAO/WHO recommendations among all other ages. Alpha-linolenic acid intake was <0.5%E in almost half of studies. Docosahexaenoic acid (DHA) or eicosapentaenoic acid + DHA intakes were below recommendations in most studies. In summary, while total fat intake was too low among younger children, SFA intake was above, especially in Europe and n-3 PUFA intake, especially DHA, were below recommendations for all ages. Intake of n-3 PUFA, especially DHA, is generally suboptimal. More data, particularly from developing countries, are required to refine these findings and guide implementation of adapted nutrition policies.

## 1. Introduction

Dietary fat has broad physiological functions. It provides essential fatty acids (EFA) required for growth and development and contributes important fat-soluble vitamins (A, D, E, K) to the diet [1]. It acts as a substrate in hormone production [2] and has been shown to play a role in visual and cognitive development [3], cardiovascular health [4], as well as immune function [5]. Additionally, dietary fat enhances the organoleptic properties of food by enhancing the taste, texture, flavour and aroma, and thus affects palatability and acceptability of these foods [6]. Dietary fats may also affect satiety by modulating gastric emptying and intestinal motility [7,8].

Dietary fat is an important source of energy during early life and plays an essential role in supporting rapid growth and development during this period [1]. During childhood, requirements for specific fatty acids such as polyunsaturated fatty acids (PUFA) are higher than at other times in life [9]. Important dietary fatty acids (FA) include the two EFA (linoleic [18:2n-6, LA] and alpha-linolenic acid [18:3n-3, ALA]), as well as the long-chain derivatives of ALA, eicosapentaenoic (20:5n-3, EPA) and docosahexaenoic acid (22:6n-3, DHA) [1].

Given the critical role of dietary fats in child growth, development and health, various health authorities have generated fatty acid intake recommendations specific for children. These recommendations differ according to children’s needs and thus, according to age. Indeed, given that the requirements for fat and certain PUFA are relatively high in children, the younger population is at greater risk for inadequate intake. Moreover, early life dietary habits and dietary intake from the complementary diet and school or family meals may not cover the needs for specific fatty acids for these age groups [1,10].

Many existing studies have explored fat and fatty acid intakes and imbalances in children [11,12] and several reviews highlight inadequate intake of certain FA (particularly PUFA) among children when compared to the recommendations [13,14,15,16]. However, most of the reviews have grouped and analyzed together the intakes of children of quite different ages, e.g., less than 10 years [14,15] or less than 5 years [16]. Two of these reviews assessed observed intake against WHO 2003 reference nutrient intakes (RNIs) for adults [14,15], one against Institute of Medicine RNIs for children from 2002 [16] and one against the European Food Safety Authority (EFSA) 2010 dietary reference values (DRVs) for children [13]. While two studies are limited to children from Europe [13,14] and one from the Eastern Mediterranean region [16], another included intake data reported globally [15]. Lastly, only two [13,15] reported intake of individual PUFA.

The aim of this review is to gather the available literature on the intake of total fat, SFA and individual PUFA in children across different age ranges: 1–2 years, 2–3 years, 3–5 years, and 5–7 years worldwide and to compare data obtained against the most recent and age-appropriate fat recommendations. Firstly, an overview of the most recent recommendations for intake of fat according to two international health authorities, the Food and Agriculture Organization/World Health Organization (FAO/WHO) [17] and EFSA [18,19]) within these age ranges is given. Secondly, the available intake data in children aged 1 to 7 years are summarised and reported, and the gaps between reported intake and recommendations according to the FAO/WHO and EFSA are highlighted and discussed for each age group.

## 2. Materials and Methods

### 2.1. Evaluation of Current Recommendations for Intake of Total Fat and Specific Fatty Acids

The most recent international dietary reference intakes (DRIs) for fats: FAO/WHO Acceptable Macronutrient Distribution Range (AMDR) and EFSA Adequate Intake (AI) were recovered and tabulated. These DRIs are expressed as a percentage of energy (%E) except for DHA and EPA + DHA, which are expressed in absolute weight. The following age segmentation was used: 1–2 years (12–<24 months), 2–3 years (24–<36 months), 3–5 years (36–<60 months) and 5–7 years (60–<84 months). The age ranges were defined in accordance with the age ranges used by the two authorities to express the DRIs, and to allow more granularity in assessing the data of school children.

### 2.2. Evaluation of Current Intakes for Total Fat and Specific Fatty Acids

#### 2.2.1. Search Methods for Identification of Studies

The literature search was conducted independently by two researchers using Medline and Google Scholar electronic databases from April–June 2021. No limit was placed on the date of publication of the retrieved studies. Key words for the search were “lipids”, “fat”, “fat intake”, “nutrients”, “nutrient intake”, “children”, “toddlers”, “saturated fatty acids”, “linoleic”, “linoleate”, “18:2n-6”, “linolenic”, “linolenate”, “18:3n-3”, “DHA”, “docosahexaenoic”, “docosahexaenoate”, “22:6n-3”, “EPA”, “eicosapentaenoic”, “eicosapentaenoate”, “20:5n-3”. Inclusion criteria were as follows: full text articles with abstracts in English or in French; presence of intake data for SFA or at least one specific PUFA (LA, ALA or DHA); inclusion of children in the age range of 1 to 7 years; from any country; national, subnational or local intake survey; intervention studies with data at baseline or a control group; dietary assessment method validation studies. There was no limit placed on time of dietary assessment, however, when more recent data were available for the same FA and the same population, only the more recent data were reported. Because of the paucity of data in some countries, or for some FA or some age ranges, results of small (n = 25–100 subjects) studies were also included. Reference lists of review articles were checked for additional, relevant studies, and values from review articles were included when no original paper was found. Data from national surveys and certain national agencies were also searched, for which no language filter was applied. Studies focused on children with diseases or children with specific dietary habits were excluded.

#### 2.2.2. Data Selection

Articles identified through the search were screened and irrelevant articles were excluded based on their title, then on their abstract. The full text of all relevant articles was then assessed for eligibility.

#### 2.2.3. Data Extraction

The following standardised information was extracted from each study and compiled into one large database organized by geographical continent: country, study name, study type, name of first author, year of publication, year(s) of data collection, participant demographics (age, gender, private/public school or income level), number of participants (n), method used for dietary assessment; intake data extracted were means and/or medians for total energy (kJ/day and kcal/day), total fat (g/day and total daily energy percentage [E%]), total SFA (g/day and E%), LA and/or total n-6 PUFA (g/day and E%), ALA and/or total n-3 PUFA (g/day and E%), DHA (mg/day), EPA + DHA (mg/day), other FA (total MUFA, total PUFA, total LC n-3 PUFA, docosapentaenoic acid (22:5n-3, DPA) or arachidonic acid (20:4n-6, ARA). For studies that provided data with and without dietary supplements, data including supplements were reported. When the same dietary survey was used to report (or calculate) different outcomes (e.g., different fatty acids) in several papers, data for each paper were kept separate and counted as a separate study. When several dietary studies were available for one country, all were included when they covered different populations (age groups or representativeness).

#### 2.2.4. Data Assessment

Mean or median fatty acid intake data were evaluated against the FAO/WHO and EFSA recommendations using the same segmentation as outlined above: 1–2 years, 2–3 years, 3–5 years and 5–7 years [17,18,19,20]. Data evaluated included the fats for which intake recommendations were found, namely total fat, total SFA, LA, ALA, DHA, and the sum of EPA and DHA. The comparison with the recommendation was done using the following units: contributions to total daily energy intake (%E) for total fat, SFA, LA and ALA; absolute values (mg/day) for DHA and EPA. Because the age range classification differed between papers, we included some of the reported data in more than one of the four age groups of interest (e.g., intake data for children 1 to 3 years of age were evaluated against the recommendations for children 1 to 2 years and that of children 2 to 3 years). For studies where the age range extended beyond our inclusion criteria, data were reported only for our relevant ages (e.g., studies reporting intake for children age 2–8 years were included in the 2–3 years, 3–5 years and 5–7 years groups). The number and proportion of studies with intake outside the recommendations were then calculated. In studies that reported data for two groups (e.g., mean for boys and mean for girls) each of the two sub-groups were counted as half a study. Due to the substantial heterogeneity of the included studies, meta-analysis of the data was not possible.

## 3. Results

### 3.1. Intake Recommendations for Total and Specific Fatty Acids

The FAO/WHO and the EFSA DRIs for fats and FA for children 1–7 years of age are summarized in Table 1. The table provides the DRI relative to the total daily energy intake (%E) (total fat, total SFA, LA, ALA, total PUFA, total MUFA) and in mg per day (DHA and sum of EPA+ DHA) for children within the following age ranges: 1–2 years, 2–3 years, 3–5 years and 5–7 years.

#### 3.1.1. Total Fat Intake Recommendations

The DRI used by the two authorities is the AMDR, i.e., the range of total fat intake associated with reduced risk of chronic disease while providing an adequate fat intake. The values are based on evidence that the consumption above or below these ranges may be associated with nutrient inadequacy and increased risk of developing chronic diseases (coronary heart diseases [CHD], obesity, diabetes, and/or cancer). The recommendations are to have a gradual reduction in the fat contribution to the total daily energy intake from 6 months of age depending on the physical activity level of the child, by age 3 years to approximately 30–35% of energy [1] or by age 4 years to 20–35% of energy [18], in line with the upper adult AMDR.

#### 3.1.2. Total Saturated Fatty Acid Intake Recommendations

The FAO/WHO [17] sets a numerical value, as an upper-AMDR in view of limiting the risk of CHD. Currently, it recommends a maximum of 8 E% for total SFA for children (2–18 years); however, new WHO guidelines are in preparation and it seems, from the available draft [21], that the value for children will be aligned to the adult’s value of 10%E.

EFSA [18] concluded that SFA can be synthetized by the body and so are not required in the diet. They recognized a positive, dose-dependent relationship between the intake of a mixture of SFA and blood LDL cholesterol concentrations (risk factor for cardiovascular disease) when compared to carbohydrates and finally advised a SFA intake “as low as possible”. It should be noted that the value for adults was applied to children without providing a rationale. Knowing that the contribution of SFA to breast milk lipids is considerable (35–40%) [22] and that the WHO recommends continued breastfeeding until 2 years of age [23], more guidance is needed in Europe.

A more flexible approach has been taken by other bodies, such as the Scientific Advisory Committee on Nutrition (SACN). The UK recently released [24] its dietary reference value for SFA, unchanged from the previous one issued in 1994 [25], which was “no more than about 10%”. However, this recommendation does not apply before 2 years of age and applies in full from 5 years and into adulthood [25]. A flexible approach was recommended in relation to the timing and extent of dietary change for individual children between 2 and 5 years.

#### 3.1.3. N-6 PUFA—Linoleic and Arachidonic Acid Intake Recommendations

FAO/WHO [17] proposed for LA an estimated average requirement (EAR), i.e., the intake level at which the needs of 50% of the population are met, of 2%E and an AI of 2–3%E after two years of age, based on the fact that the signs of deficiency can be prevented by providing 1–2%E LA in adults. The higher value of the AI (i.e., 3%), as part of a healthy diet, is contributing to long term health by lowering LDL and total cholesterol levels and consequently the risk for CHD. The AI for younger children aged 1–2 years is higher (3–4.5%E). No recommendation was set for ARA.

EFSA [18,19] set an AI for LA, because they estimated there was no evidence to support an EAR. The AI for LA for all populations was based on the lowest estimated mean intakes of the various population groups in Europe, where overt LA deficiency symptoms were not present. For children aged 1–3 years old, they noted that LA intake was generally below the adult AI of 4 E%, but that no signs of LA deficiency were observed if LA intakes were >1 E%, so that intake and status of LA in infants and young children living in Europe were of no concern. The adult AI of 4%E was proposed for children. While acknowledging that ARA was present in human milk, EFSA did not identify clinical effects of supplementing infants or children with ARA [26,27], and thus did not set any recommendation for this fatty acid. This controversial recommendation is not supported by all experts in the field [28].

#### 3.1.4. N-3 PUFA: Alpha-Linolenic Acid and EPA + DHA Intake Recommendations

The FAO/WHO [17] concluded that the ALA minimum dietary requirement for adults (lower AMDR: >0.5%E) does prevent deficiency symptoms, while the highest value (upper AMDR, 2 or 3%E, depending on the age) can be part of a healthy diet. FAO/WHO set an AI for DHA up to the age of 2 years because of its critical role in retinal and brain development and, after the age of 2 years, an AI for EPA + DHA as part of a healthy diet in view of preventing the risk of chronic diseases (total CHD events, stroke). This AI is age adjusted up to the age of 10 years, after which the adult value applies. FAO/WHO also mentions that a specific minimum to prevent n-6 and n-3 PUFA deficiency is unclear.

Similar to LA, EFSA [18,19] found that the mean ALA intakes were below the AI (0.5%E) in some populations of young children in Europe. However, there is no clear relationship between intakes or biomarkers of n-3 PUFA status and clinical outcomes. Thus, EFSA concluded that they could not quantify the risk of inadequate intakes of ALA and DHA in young children living in Europe. Given that DHA is needed for the normal development of the nervous system and the retina, and accumulates in the brain and retina during early childhood, they considered DHA as a conditionally essential FA for infants. The AI of 100 mg DHA per day for the age range 6–24 months was based on “intervention studies with DHA-enriched formula or complementary foods from age 1.5 to 12 months in formerly and continuing breast-fed infants and which was found to be effective for visual function”. After the age of 2 years, the same AI as for adults was proposed (250 mg EPA + DHA/day), an amount considered as enough for primary prevention of CHD in healthy subjects [18].

#### 3.1.5. Comparison between FAO/WHO and EFSA Fat Recommendations

The DRIs for fats do not differ much between the two authorities. Nevertheless, compared to FAO/WHO, EFSA has set higher minimum values for LA (4%E vs. 2 or 3%E) and for EPA + DHA for children older than 2 years (250 mg/day vs. 100 mg/day [2–3 years], 125 mg/day [3–5 years] or 175 mg/day [5–7 years]). EFSA has made age-specific recommendations for total fat (until the age of 4 years) and DHA (until the age of 2 years) while FAO/WHO values were age-specific for LA also and evolved until the age of 18 years. Most recommendations are expressed as %E, so the recommended absolute amounts of fat and PUFA increase with age, according to the increasing energy intake. The FAO/WHO also set an upper AMDR for total PUFA (15%E for the age 1–2 years and 11% for 2–18 years) and an AMDR for MUFA (obtained by difference between total fat and all the other FA types). EFSA did not set any DRV for total PUFA or total MUFA. Despite its presence in human milk and its role in building structures of all body cells, no recommendation has been set for the intake of ARA.

### 3.2. Effective Intake for Total and Specific Fatty Acids

#### 3.2.1. Data Extracted: Reported Intakes of Total and Specific Fatty Acids

After initial consideration, several articles were excluded from the final selection: Butte et al., [29] for the USA, Murakami et al., [30] for Japan, Ocké et al., [31] for The Netherlands and Meyer [32] for Australia because more recent data were available from the new national surveys (Bailey [33] for the USA, NHNS-J 2018 for Japan [34], van Rossum for the Netherlands [35] and Meyer [36], Rahmawaty [37] and Rangan 2014 [38] for Australia. Finally, we identified 65 studies including children from age 1 to 7 years, done in 33 countries, published between 2000 and 2021. Several studies were identified from North America, Asia, Europe and Oceania, while there were very few studies from Africa and South America. A summary of all the studies collected can be found in Table 2. The number of studies recovered per geographical area is as follows (with number of studies per country in parentheses):

- Three in Africa: Gambia, South Africa (two).
- Twelve in North America: Canada (six), USA (four), Mexico (two)
- Three in South America: Argentina, Brazil, Chile
- Fifteen in Asia: Bangladesh, China, Indonesia, Japan (four), South Korea (two), Malaysia, Philippines (two), Singapore (three)
- Twenty-five in Europe: Belgium (two), Cyprus, Finland (three), France (two), Germany (two), Greece, Ireland (two), Italy, Poland, Spain (two), Sweden, The Netherlands (two), UK (four), Turkey
- Two in Middle East: Lebanon, United Arab Emirates (UAE)
- Five in Oceania: Australia (five)

Fifty of the 65 included studies reported data from high income countries. Thirty-eight of the 65 studies were based on national studies, while 27 were either local or subnational studies (Table S1). Most studies included more than 100 subjects, but in 10 studies, e.g., two of the three studies done in Africa, the number of subjects per age group was less than 100. Data were collected largely in the last 10 years, but for some geographies, e.g., Africa, data were quite old (collected in 1978–1980 for Gambia, in 1998 in one of the two South African studies). While the assessment of fat intake was generally not the objective of the studies, 22 studies mentioned fat as one of their objectives (Table S1). These included 13 studies targeting PUFA, five of them specifically assessing n-3 PUFA. Only 23 studies in total reported DHA intake. The methods used to assess dietary intake were diverse (Table S2). Twenty-two studies recorded intake using dietary recall (10 used 24 h recall once; six used a 24 h recall at least twice and six used a 24 h dietary recall on whole sample + x days dietary recall on a subsample), 28 used a food record (mostly 3 day or more diary), while the remaining dietary assessment methods were either food frequency questionnaires (7), whole diet collection chemical analysis (one study) or combinations of several methods. Most studies did not inform on whether fortified foods or supplements were included. Nineteen studies reported they included fortified foods and 21 reported they included supplements (Table S3). Most of these supplements were vitamins and minerals, while only a few studies specifically reported that they included PUFA oil or fish oil supplements in their dietary assessment [35,36,39,40,41,42]. Thirteen of the 31 studies done in 1–2 year-old children indicated that breast milk intake was included as part of the children’s intake, and six reported that it was not, while 12 did not provide the information (Table S3).

Table 2 reports the observed daily intake of energy, total fat, total SFA, LA and/or total n-6 PUFA, ALA and/or total n-3 PUFA, DHA, EPA + DHA in children 1–7 years old. The last column reports unsaturated FA, which are not in the scope of our review because there was no (ARA, DPA, total n-3 long chain-PUFA [LC-PUFA]) or only limited (total MUFA or total PUFA) intake recommendations. Table 2 values represent means (when not specified) or medians of a variable number of children.

**Table 2:** Observed daily energy, total fat and specific fatty acids intake in 1–7 years old children [means (SD), when not specified, or median values].

Country Year Data Collect. Study Type	Ref.	Dietary Method	Population Age, Gender, n	Energy kJ/kcal	Total Fat (%E or g)	SFA (%E or g)	LA (%E or g)	ALA (%E or mg)	DHA (mg)	EPA + DHA (mg)	Other FA
Africa											
The Gambia (rural, one village) 1978–80	Prentice 2000 [43]	>1 d Record	1–2 y 13–17 Mon. n = 115	3673 kJ (878 kcal) ^1^	~27.5%E ^2^ 26.85 g	~8.7%E 8.49 g	5.1%E 4.95 g	0.23%E 220 mg	80	NR	MUFA: 12.75 g n-6 PUFA: 5.26 g n-3 PUFA: 380 mg PUFA: 5.61 g ARA: 60 mg
			2 y 24 Mon. (=after weaning) n = 42	3263 kJ (780 kcal)	~15%E 13 g	~3.6%E 3.16 g	4.6%E 3.99 g	0.13%E 120 mg	10	NR	MUFA: 5.86 g n-6 PUFA: 4 g n-3 PUFA:140 mg PUFA: 4.09 g ARA: 10 mg
S. Africa 1 (2 similar informal household settlements in Mangaung: Joe Slovo, JB Mafora) 1998 Cross-sectional survey	Dannhauser 2000 [44]	24 h	2–3.9 y JS n = 63 JB n = 68	JS 3424 kJ (1489) ~818 kcal (355.8) JB 4274 kJ (1653) ~1022 kcal (395.1) Medians: JS 3246.78 kJ ~776 kcal JB 3995.72 kJ ~955 kcal	JS ~26.4%E 24 g (12.2) JB ~26%E 29.5 g (19.1) Medians: JS 23.7 g JB 15.3 g	JS ~9.6%E 8.7 g (5) JB ~8.7%E 9.9 g (6.7) Medians: JS 8.2 g JB 8.0 g	“n-6 EFA” JS ~5.5%E 5.0 g (4.2) JB ~6.5%E 7.4 g (7.2) Medians: JS 3.7 g JB 2.9 g	“n-3 EFA” JS ~0.44%E 400 mg JB ~0.35%E 400 mg Medians: JS 300 mg JB 200 mg	NR	NR	PUFA: JS 5.4 g (4.2) JB 7.8 g (7.1) Medians: JS 4.2 g JB 5.9 g
			4–5.9 y JS n = 46 JB n = 54	JS 3780 kJ (1610.8) ~903 kcal (384.9) JB 4383 kJ (1560.6) ~1048 kcal (372.8) Medians: JS 3535.4 kJ ~845 kcal JB 4184 kJ ~1000 kcal	JS ~25.3%E 25.4 g (18.1) JB ~23.8%E 27.7 g (17.3) Medians: JS 22.5 g JB 24 g	JS ~9.0%E 9.0 g (5.4) JB ~7.9%E 9.2 g (5.8) Medians: JS 8.6 g JB 8.9 g	“n-6 EFA” JS ~6.2%E 6.2 g (7.3) JB ~5.8%E 6.7 g (5.6) Medians: JS 3.8 g JB 5.4 g	“n-3 EFA” JS ~0.30%E 300 mg (300) JB ~0.26%E 300 mg (300) Medians: JS 200 mg JB 300 mg	NR	NR	PUFA: JS 6.5 g (7.2) JB 7.1 g (5.6) Median JS 4.1 g JB 5.8 g
S.Africa 2 2 provinces (mixed urban and rural) (PDIS) 2018–2019 Cross sectional survey	Steyn 2020 [45]	24 h + 2x (24 h)	1–<3 y n = 333	Mean (SE) ^3^ 4944 kJ (244.6) ~1182 kcal (58.5)	30.3%E (0.9) 39.4 g (2.3)	8.9%E (0.7) 12 g (1)	NR	NR	NR	NR	MUFA: 9.0%E (0.7) 11.4 g PUFA: 7.8%E (0.5) 9.5 g
			3–<6 y n = 514	5626 kJ (184.4) ~1345 kcal (44.1)	28.7%E (0.6) 44 g (2.6)	8.5%E (0.3) 13.1 g (1.2)	NR	NR	NR	NR	MUFA: 9%E (0.2) 13.7 g PUFA: 8.1%E (0.2) 12.2 g
America North											
Canada 1 National (CCHS) 2015 Cross-sectional survey	Barr 2018 [46]	24 h	6–12 y (breakfast eaters) n = 2227	Mean (SE) ~7757.14 kJ (92) 1854 kcal (22)	30.1 (0.3)%E 64 (1.0) g	~10.9%E 23.1 (0.4) g	NR	NR	NR	NR	MUFA:~11%E; 22.7 (0.4) g PUFA: ~6%E; 12.4 (0.3) g
Canada 2 National (CCHS) 2004 Cross-sectional survey	Health Canada 2012 [47,48]	24 h	1–3 y n = NR	~6700 kJ ^4^ 1611 kcal	30.3%	12%E	NR	Median ~0.47% 840 mg	NR	NR	MUFA: 11%E PUFA: 4%E
			4–8 y n = NR	~7995 kJ ^4^ 1911 kcal	30.1%	12%E	NR	Median ~0.49% 1023 mg	NR	NR	MUFA: 11%E PUFA: 4%E
Canada 3 Vancouver 2003 Cross-sectional study	Innis 2004 [49]	FFQ Total n = 84	1.5–2 y (18–24 Mon.) n = NR	Mean (SE) 7327 kJ (509) ~1751 kcal (120)	32(1.83)%E	26.7 g (3.09)	~2.98%E 5.80 g (0.57)	~0.60%E 1160 mg (160)	41 (10)	~70 ^5^	MUFA: 21.4 g (2.36) PUFA: 7.58 g (0.67) EPA: 29 mg (8) ARA: 133 mg (21)
			2–3 y (25–36 Mon.) n = NR	Mean (SE) 9619 kJ (707) ~2299 kcal (169)	34.6(1.53)%E	~13.3%E 35.7 g (3.67)	~3.5%E 9.04 g (0.63)	~0.79%E 2020 mg (230)	95 (16)	~152 ^5^	MUFA: 30.9 g (2.83) PUFA: 11.7 g (0.84) EPA: 57 mg (11) ARA: 260 mg (39)
			3–5 y (37–60 Mon.) n = NR	Mean (SE) 9518 kJ (426) ~2278 kcal (102)	32.4(0.75)%E	12%E 30.4 g (1.67)	~3.7%E 9.39 g (0.63)	~0.70%E 1720 mg (170)	96 (14)	~156 ^5^	MUFA: 29.9 g (1.86) PUFA: 11.3 g (0.74) EPA: 60 mg (8) ARA: 226 mg (17)
Canada 4 Alberta (e.g., far from coastal) 2001–2002 Cross-sectional study	Lien 2009 [50]	3 d Record	4–7 y n = 78	~7363.8 kJ (1840.96) 1760 (440) kcal Median ~7238 kJ 1730 kcal	33%E (4.5) 63 g (17) Median 33%E 63 g	12%E (2.4) 23 g (7.9) Median 12%E 22 g	3.9%E (1.6) 7.4 g (3.3) Median 3.7%E 6.8 g	0.36%E (0.20) 710 mg Median 0.35%E 620 mg	37 (63) Median 16.5	~54	MUFA: 12%E (2.3) = 22 g (6.1) Medians: 11%E = 22 g n-6 PUFA: 7.4 g (3.3) Median: 6.7 g n-3 PUFA: 750 mg (500) Median: 720 mg PUFA: 5.1%E (1.6) = 9.8 g (3.6) Medians: 5%E = 9.4 g ARA: 57 mg (30.5) Median: 51 mg EPA: 17 mg (30.6) Median 4.6
Canada 5 Ontario 2009 Survey type not reported	Madden 2009 [51]	Chemical analysis (3 d food collection aliquots)	4–8 y n = 41	Mean (SEM) 5879 kJ (209) ~1405 (50) kcal	22.8%E (0.9) 36.3 g (2.5)	8.4%E 13.1 g (0.7) Median 13 g	4.9%E 7.6 g (0.7) Median 6.3 g	0.74%E 1161 mg (108) Median 1007 mg	54.1 (11.4) Median 21.9	92.5 (20.2) Median 31.5	MUFA: 9.3%E,14.6 g n-6 PUFA 5%E, 7.7 g (0.7) n-3 PUFA 0.83%E, 1298 mg (119) PUFA: 5.8%E, 9.0 g ARA: 61.7 mg (5.8) Median 58.5 mg EPA: 38.4 mg (9.3) Median 12.0 mg DPA: 26.3 mg (3.9) Median 18.8 mg
Canada 6 Vancouver 2010–2014 Secondary analysis of DBRCT	Wiedeman 2020 [52]	3 d Record	1 y (12–14 Mon.) n = 110	NR	NR	NR	3.74%E (1.52) 3.95 g (1.78) Median 3.42%E 3.63 g	0.53%E 0.26) 560 mg (290) Median 0.47%E 500 mg	31. (58.7) Median 10	47. (100) Median 10	EPA: 16 mg (42.2) Median 0 DPA: 3.52 mg (9.72) Median 0 ARA: 36.8 mg (30.4) Median 30 mg
			2 y n = 86	NR	NR	NR	4.2%E (1.68) 5.19 g (2.63) Median 3.96%E 4.3 g	0.59%E (0.31) 740 mg (490) Median 0.49%E 580 mg	64.8 (107) Median 20	100 (177) Median 20	EPA: 35.8 mg (72.7) Median 0 DPA: 9.42 mg (19.6) Median 0 ARA: 52.7 mg (36.2) Median 45 mg
Mexico 1 National (ENSANUT) 2012 Cross sectional survey	Jiménez-Aguilar 2018 [53]	7 d semi-quantitative FFQ	1–2 y (12–35 Mon.) n = 593	Median ~4585.66 kJ 1096 kcal	Median ~32.1%E 39.1 g	Median ~14.6%E 17.8 g	NR	NR	NR	NR	Median PUFA: ~5.7%E 7 g
			3–4 y (3–59 Mon.) n = 619	Median ~5669 kJ 1355 kcal	Median ~32.2%E 48.5 g	Median ~14.2%E 21.4 g	NR	NR	NR	NR	Median PUFA: ~6%E 9 g
Mexico 2 National (ENSANUT) 2006 Cross sectional survey	Ramirez-Silva 2011 [54]	7 d FFQ	5–11 y n = 8690	Mean (SE) ~5840.8 kJ (49.8) 1396 kcal (~11.9)	26.7%E (0.16) 39.5 g (0.5)	11.4%E (0.08) 15.6 g (0.2)	~3.2%E 4.4 g (0.05)	~0.2%E 300 mg (50)	NR	NR	MUFA: 9.4%E (0.06) 13.1 g (0.15) n-6 PUFA: 3.3%E 4.5 g (0.05) n-3 PUFA: 0.2%E ~300 mg (10) PUFA: 5.9% (0.04) = 8.2 g (0.10)
USA 1 National (NHANES) 2009–2012 Cross-sectional survey	Ahluwalia 2016 [55]	2x 24 h + 1x (24 h)	1–2 y (12–23 Mon.) n = 516	Mean (SE) ~4995.7 kJ (103.3) 1194 (24.7) Median: ~4916.2 kJ 1175 kcal	32.9 (0.4)%E 44.1 (1) g Median: 33% = 43.1 g	~13.6%E = 18.0 (0.5) g Median: 17.5 g	~4.98%E = 6.6 (0.2) g Median: 6.3 g	~0.65%E = 860 (20) mg Median: 830 mg	NR	NR	MUFA: 14.5 (0.5) g Median: 14.1 g PUFA: 7.6 (0.3) g Median: 7.2 g
USA 2 National (FITS) 2016 Cross-sectional study	Bailey 2018 [33]	24 h + 1x (24 h)	1–2 y (12–23.9 Mon.) n = 1133	Mean (SE) ~4891.1 kJ (37.6) 1169 kcal (9) Median: ~4732 kJ 1131 kcal	33 (0.1)%E = 44 ± 0.4 Median: 33%E = 42 g	13 (0.1)%E = 18 ±0.2 g Median: 13%E = 17 g	NR	NR	NR	NR	NR
			2–3 y (24–35.9 Mon.) *n* = 305	~5769.7 kJ (87.86) 1379 kcal (21) Median: ~5594 kJ 1337 kcal	31 (0.3)%E = 49 ± 0.9 Median: 31%E = 47 g	11 (0.2)%E = 18 ± 0.4 g Median: 11%E = 17 g	NR	NR	NR	NR	NR
			3–4 y (36–47.9 Mon.) *n* = 295	~5920 kJ (92.0) 1415 (22) Median: ~5732 kJ 1370 kcal	31 (0.3)%E = 50 (0.9) g Median: 31%E = 48 g	11 (0.2)%E = 18 (0.4) g Median: 11%E = 17 g	NR	NR	NR	NR	NR
USA 3 National (MEC component of NHANES) 2003–2008 Cross-sectional study	Keim 2015 [56]	24 h	1–2 y (17–24 Mon.) n = 802	NR	NR	NR	Mean (SE) ~>5.5%E ^6^ 7.03 (0.290) g	~0.65%E ^6^ 820 (33) mg	19.78 (3.00)	~24.78	n-6 PUFA: 6.97 (0.29) g n-3 PUFA: 940 (30) mg ARA: 56.13 (3.24) mg EPA: 5 (0.56)
			2–3 y (25–36 Mon.) n = 858	NR	NR	NR	~>5.5%E ^7^ 7.99 (0.283) g	~0.59%E ^7^ 860 (32) mg	20.50 (3.03)	~26.22	n-6 PUFA: 8.19 (0.3) g n-3 PUFA: 890 (35) mg ARA: 56.87 (2.71) mg EPA: 5.72 (0.63) mg
			3–4 y (37–48 Mon.) n = 454	NR	NR	NR	~6.2%E ^7^ 9.01 (0.315) g	~0.59%E ^7^ 870 (34) mg	20.80 (3.20)	~27.21	n-6 PUFA: 9.10 (0.33) g n-3 PUFA: 900 (38) mg ARA: 61.64 (3.96) mg EPA: 6.41 (0.70) mg
			4–5 y (49–60 Mon.) n = 496	NR	NR	NR	~6%E ^8^ 10.16 (0.40) g	~0.53%E ^8^ 900 (39) mg	21.00 (3.59)	~28.18	n-6 PUFA: 10.16 (0.40) g n-3 PUFA: 930 (43) mg ARA: 67.63 (4.38) mg EPA: 7.18 (0.82) mg
USA 4 National (NHANES) 2003–2014 Cross-sectional survey	Thompson 2019 [39]	2x 24 h	1–5 y n = 5392	NR	NR	NR	NR	NR	Mean (SE) ~19.5–25 mg ^9^ 16.1 (0.6) mg/1000 kcal	~24.7–35.4 mg ^9^ 23.1 (0.6) mg/1000 kcal	NR
			6–11 y n = 5550	NR	NR	NR	NR	NR	~34 mg ^10^ 17.5 (0.9) mg/1000 kcal	~50.8 mg ^10^ 25.9 (1.4) mg/1000 kcal	NR
America South											
Argentina Buenos Aires province 2013 Cross-sectional study	Lazaro-Cuesta 2018 [57]	24 h	6–8 y n = 387	Medians M ~8681.8 kJ 2075 kcal F ~8468.4 kJ 2024 kcal	Medians M ~37.6%E 86.7 g F ~36.7%E 82.5 g	Medians M ~13.1%E 30.2 g F ~11.4%E 25.6 g	NR	NR	NR	NR	Medians MUFA: F 34.4 g; M 30.8 g PUFA: F 22.3 g; M 17.6 g
Brazil 9 cities 2007 Cross-sectional study	Bueno 2013 [58]	1 d Record + 1x (1 d Record)	2–3 y public school (n = 1278) private school (n = 425)	Mean (SD or SE [not specified]) ~6945.4 kJ (1551) 1660 kcal (371) ~6861.8 kJ (1647.2) 1640 kcal (394)	28.1 (5.8)%E 28.5 (5.5)%E	9.9 (2.2)%E 9.9 (2.4)%E	NR	NR	NR	NR	NR
			4–6 y public school (n = 1041) private school (n = 314)	~7066.8 kJ 1689 kcal (364.4) ~6962.2 kJ 1664 kcal (357.3)	28.6 (5.7)%E 28.8 (5.4)%E	9.8 (2.1)%E 9.8 (2.2)%E	NR	NR	NR	NR	NR
Chile South-East Santiago (FECHIC cohort) 2016 Cohort study	Rebolledo 2019 [59]	24 h + 1x (24 h)	3–6 y n = 839 (low and middle income)	Mean (SE) ~5259.3 kJ (56.9) 1257 kcal (13.6)	NR	9.9 (0.1)%E 13.9 (0.2) g	NR	NR	NR	NR	NR
Asia											
Bangladesh 2 rural districts 2007–2008 Cross-sectional survey	Yakes 2011 [60]	2x (24 h) + correction	2–3 y (24–35 Mon.) n = 221	Mean (SE) ~3426.7 kJ 819 kcal (~89)	17:2 (6.1)%E 15.2 (5.5) g	~5.7%E 5 (2.5) g	3.3 (1.4)%E 3 (1.4) g	0.4 (0.16)%E 370 (168) mg	30 (20)	NR	MUFA: 5.1 (2.0) g PUFA: 3.5 (1.7) g ARA: 60 (58) mg
			3–4 y (36–48 Mon.) n = 236	~3895.3 kJ 931 kcal (72)	12.9 (5.2)%E 13.5 (6) g	~3.4%E 3.6 (1.9) g	2.9 (1.3)%E 3.1 (1.6) g	0.41 (0.18)%E 430 (200) mg	20 (5)	NR	MUFA: 4.8 (2.2) g PUFA: 3.6 (1.9) g ARA: 50 (31) mg
China Yunnan province (rural) 2003–2004 Cross-sectional survey	Barbarich 2006 [61]	3x 24 h	1–3 y n = 126	~2761.4 kJ (1079) 660 kcal (258)	24 (7)%E 18 (10) g	NR	2.9 (1.2)%E 2.08 (1.17) g	0.4 (0.3)%E 278 (229) mg	34 (148)	NR	n-6 PUFA: 2.2 (1.3)%E 2.14 (1.24) g n-3 PUFA: 0.5 (0.4)%E 366 (343) mg ARA: 55 (42) mg
			4–5 y n = 70	~3418.3 kJ (933) 817 kcal (223)	21(7)%E 19 (9) g	NR	2.5 (1.1)%E 2.27 (1.22) g	0.4 (0.3)%E 335 (232) mg	23 (87)	NR	n-6 PUFA: 2.5 (1.1)%E 2.27 (1.22) g n-3 PUFA: 376 (319) mg ARA: 23 (87) mg
Indonesia National (RISKESDAS) 2010 Cross sectional	Neufingerl 2016 [62]	1x 24 h	4–8 y n = 25,417	Median 4398 kJ ~1050 kcal	Median 27.2%E 31.2 g	Median 11.6%E 13.3 g	Median 3.3%E 4.0	Median 0.20%E = 245 mg	NR	Median 30 mg	Median PUFA: 4.0%E 4.7 g
Japan 1 Seiiku Boshi cohort 2010–2013 Cohort study	Ando 2019 [63]	1 Mon. FFQ	3 y n = 153	3399.9 kJ (906.3) ~812.6 kcal (217)	NR	NR	n-6 PUFA ^11^ ~4.7%E 5.0 g (1.5)	~>0.5%E ~540 mg (estimated) ^12^	98.3 (64.6)	148.4 (106)	n-3 PUFA: 0.9 g (0.3) PUFA: 5.8 g (1.7) ARA: 53.5 mg (21.4) EPA: 49.4 (43.5) mg
Japan 2 National (NHNS-J) 2012 Cross sectional study	Murakami, 2018a [30]	1 d Record	> = 1–6 y (12–83 Mon.) n = 1289	Mean (SE) 1278 kcal (10.3)	28.3 (0.2)%E	9.2 (0.1)%E	NR	NR	NR	NR	MUFA: 9.8 (0.1)%E PUFA: 5.4 (0.1)%E
Japan 3 Nationwide (DONGuRI) 2015 Cross sectional study	Murakami 2018b [64]	3 d Record	Volunteer children attending nursery facilities 3–5 y (36–71 Mon.) M: 143 F: 143	Mean (SE) M 5761 kJ (908) ~1377 kcal (180) F: 5414 kJ (920) ~1294 kcal (179) Medians M 5699 kJ ~1362 kcal F 5435 kJ ~1299	M: 29.3%E (2.3) = 45.3 g (6.1) F: 29.2%E (2.2) = 42.6 g (7.0) Medians M 29.2%E = 44.7 g F 29.3%E = 41.7 g	M 9.9%E (1.4) = 15.2 g (2.5) F 9.6%E (1.2) = 14.1 g (2.5) Medians M 9.8%E = 14.8 g F 9.6%E = 13.7 g	n-6 PUFA ^11^ 4.3% M, 4.5% F M 6.7 g (1.0), F 6.5 g (1.2) Medians: M 6.7 g F 6.3 g	~ > 0.5%E ~840 mg (estimated) ^12^	NR	NR	n-3 PUFA M 1.4 g (0.3), F 1.4 g (0.3) Medians: M 1.4 g, F 1.4 g
Japan 4 National (NHNS-J) 2005–2009 Cross-sectional survey	Tsuboyama-Kasaoka 2013 [65]	1 d Record	> = 1–2 y (19–35 Mon.) M: 351 F: 338	M: ~1096 kcal ^13^ F: ~1021.75 kcal ^13^	NR	NR	n-6 PUFA ^11^ M 5.2 g (2.7) 4.27%E F 4.9 g (2.9 (~4.31%E) Median M 4.8 g; F 4.5 g	~0.52%E ~630 mg (estimated) ^12^	NR	NR	n-3 PUFA: M 1.1 g (0.7) F 1.0 g (0.7) (0.9%E) Median M 0.9 g; F 0.9 g
			> = 3–5 y (36–71 Mon.) M: 631 F: 640	M: 1428.25 kcal ^13^ F: 1288 kcal ^13^	NR	NR	n-6 PUFA ^11^ M 7.1 g (3.1) (~4.47%E) F 6.6 g (2.9 (~4.61%E) Median M 6.5 g; F 6.2 g	~0.55%E ~870 mg (estimated) ^12^	NR	NR	n-3 PUFA: Mean (SD) M 1.5 g (1.0) F 1.4 g (0.9) (~0.96%E) Median M 1.2 g; F 1.2 g
			> = 6–7 y (72–95 Mon.) M: 434 F: 453	M: 1682.75 kcal ^13^ F: 1539.25 kcal ^13^	NR	NR	n-6 PUFA ^11^ M: 8.4 g (3.6) (~4.49%E) F: 7.8 g (3.5) (~4.56%E) Median M 7.9 g; F 7.2 g	~0.67%E ~1200 mg (estimated) ^12^	NR	NR	n-3 PUFA: M 1.8 g (1.1) F 1.6 g (1.0) (~0.95%E) Median M 1.5 g; F 1.3 g
Malaysia urban areas 2010–2011 Cross-sectional study	Mohd Shariff 2015 [66]	24 h + 1x (1d Record)	1–3 y Income levels: Low (n = 72) Middle (n = 56) High (n = 53)	Mean (SE) L ~5204.9 kJ (186.6) 1244 kcal (44.6) M ~5422 kJ (215.97) 1296 kcal (51.62) H ~5050.1 kJ (221.4) 1207 kcal (52.91)	L 32.61%E (1.12) M 30.29%E (1.29) H 32.5%E (1.33)	L 13.37%E (0.48) M 12.5%E (0.56) H 11.95%E (0.57)	NR	NR	NR	NR	NR
			4–6 y Income levels: Low (n = 85) Middle (n = 89) High (n = 78)	L ~4983.1 kJ (152.3) 1191 kcal (36.41) M ~5702.8 kJ (148.4) 1363 kcal (35.47) H ~6460.1 kJ (160.2) 1544 kcal (38.28)	L 32.34%E (1.08) M 30.79%E (1.06) H 33.32%E (1.14)	L 11.88%E (0.45) M 10.68%E (0.44) H 11.65%E (0.48)	NR	NR	NR	NR	NR
Philippines 1 National (NNS) 2013	Angeles-Agdeppa 2019 [67]	24 h + 1x (24 h)	6–9 y n = 3594	1242.6 (SE = 7) Median 1184	17.8%E (0.1) = 26.1 g (0.3) Median 17.2%E = 23.1 g	~9.27%E 12.8 g (0.2) Median 10g	NR	NR	NR	NR	NR
Philippines 2 National (NNS) 2013 Cross sectional survey	Denney 2018 [68]	24 h + 1x (24 h)	1–2 y n = 714	Mean (SE) ~3225.9 kJ (50.2) 771 kcal (12) Median ~3000 kJ 717 kcal	29.6%E = 23.8 g Median 30.3% (20.3 g)	~9.9%E = 8.5 g Median 7.9 g	NR	NR	NR	NR	NR
			2–3 y n = 727	~3510.4 kJ (54.4) 839 kcal (13) Median ~3276.1 kJ 783 kcal	23.4%E = 23.3 g Median 23.1% (20.3 g)	~9.4 E% = 8.8 g Median 6.9 g	NR	NR	NR	NR	NR
			3–5 y n = 2427	~4171.4 kJ (29.3) 997 kcal (7) Median ~3970.6 kJ 949 kcal	20.2%E = 24.1 g Median 20% (21 g)	~9.9%E = 11 gMedian 8 g	NR	NR	NR	NR	NR
Singapore 1 2015–2016 Validation study	Allan 2018 [69]	2 d Record vs. FFQ (calibrated)	12–36 Mon. n = 91	Medians: Record 4608 kJ ~1101.34 kcal FFQ 4629 kJ ~1106.36 kcal	Medians: Record ~29.9%E 36.7 g FFQ ~30.26%E 37.2 g	Medians: Record ~12.09%E 14.8 g FFQ ~12.28%E 15.1 g	NR	NR	Medians: Record 100 FFQ: 120	NR	Medians: MUFA Record: 14.1 g FFQ: 14.5 g PUFA Record: 6.1 g FFQ: 6.8 g
Singapore 2 Sub-study of GUSTO Study 2011–2013 Validation study	Lim 2019 [70]	24 h + 1x (24 h) vs. FFQ	18 Mon. n = 188	Medians 24 h 3740 kJ 894 kcal FFQ 4301 kJ 1028 kcal	Medians 24 h ~30.2%E; 30 g FFQ ~28.9%E; 33 g	Medians 24 h ~6.5%E; 6.5 g FFQ ~7.0%E; 8.0 g	NR	NR	NR	NR	NR
Singapore 3 Sub-study of GUSTO Study 2015–2016 Validation study	Sugianto 2019 [71]	3 d Record (DR) vs. FFQ	5 y n = 361	Means DR ~5389 kJ (1209.2) 1288 kcal (289) FFQ ~6041.7 kJ (2129.7) 1444 kcal (509)	Medians DR ~26.5%E 37.9 g FFQ ~25.0%E 40.1 g	Medians DR ~9.1%E 13.0 g FFQ ~9.3%E 14.9 g	NR	NR	NR	NR	Medians MUFA DR 10.3 g; FFQ 12.4 g PUFA DR: 5.2 g FFQ: 5.1 g
South Korea 1 National (KNHANES VI) 2013–15 Cross sectional survey	Baek 2018 [72]	24 h	1–2 y n = 401	NR	Mean (SE) 23.3%E (0.5) 27.1 g (0.8)	Mean (SE) 9.2%E (0.3) 10.5 g (0.3)	n-6 PUFA ^11^: 3.7%E (0.1) = 4.4 g (0.2)	n-3 PUFA ^14^: 0.5%E (0.02) = 600 mg (30)	NR	NR	MUFA 7.3%E (0.2) = 8.5 g (0.3) PUFA 4.2%E (0.1) = 4.9 g (0.2)
			3–5 y n = 640	NR	Mean (SE) 23%E (0.3) 35.5(0.7)	Mean (SE) 8.3%E (0.1) 12.7 g (0.3)	n-6 PUFA ^11^: 4.0%E (0.1) = 6.3 g (0.2)	n-3 PUFA ^14^ 0.6%E (0.02) = 900 mg (40)	NR	NR	MUFA 7.3%E (0.1) = 11.3 g (0.2) PUFA 4.6%E (0.1) = 7.1 g (0.2)
South Korea 2 National (KNHANES V & VI) 2010–15 Cross sectional survey	Kim 2019 [73]	24 h	> = 1–2 y (12–24 Mon.) n = 544	NR	NR	NR	3.5%E (SEM 0.13) = 3.91 g (SEM 0.14)	0.47%E (SEM 0.02) = 529.94 mg (SEM 24.28)	37.02 (SEM 3.46)	~59.39	n-6 PUFA: 3.6%E = 4.04 g (SEM 0.14) n-3 PUFA: 0.53%E = 590 mg (SEM 30) ARA = 20.03 mg (SEM 1.52) EPA: 22.37 mg (SEM 1.77)
Europe											
Belgium 1 National 2014–2015 Cross-sectional survey	De Ridder 2015 [42]	2x 24 h + FFQ	3–5 y n = 500	~5866 kJ 1402 kcal	32.7%E	13.5%E	NR	NR	NR	NR	MUFA: 11.6%E PUFA: 5.2%E
			6–9 y n = 500	~6924.5 kJ 1655 kcal	34.6%E	13.2%E	NR	NR	NR	NR	MUFA 12.5%E PUFA 5.6%E
Belgium 2 (Flanders) 2002–2003 Cross-sectional study	Sioen 2007 [40]	3 d Record	2.5–3 y (30–48 Mon.) n = 197	NR	NR	NR	4.03%E = 6.667 g	0.48%E = 798 mg	43	~65	n-6 PUFA: 4.05%E = 6.685 g n-3 PUFA: 0.53%E = 885 mg EPA: 22 mg DPA: 10 mg ARA: 17 mg
			4–6.5 y (49–78 Mon.) n = 464	NR	NR	NR	4.25%E = 7.018 g	0.54%E = 904 mg	49	~75	n-6 PUFA: 4.24 5%E = 7.030 g n-3 PUFA: 0.58%E = 963 mg EPA 26 DPA: 10 mg ARA: 18 mg
Cyprus (non-occupied part) Nationally representative sample 2009–2010 Cross-sectional study	Tornaritis 2014 [74]	3 d Record	6–8.9 y 162 M, 158 F	M: ~7765.5 kJ (1142) 1856 kcal (273) F: ~7577.2 kJ (1138) 1811 kcal (272)	M: 34.7 (5)%E F: 34.1 (14.2)%E	M: 14 (2.7)%E F: 13.7 (2.6)%E	NR	NR	NR	NR	MUFA: M: 15.2 (3.2)%E; F: 15.1 (2.7)%E PUFA: M: 5 (1.6)%E; F: 4.8 (1.4)%E
Finland 1 DAGIS 2015–16 Cross-sectional study	Korkalo 2019 [75]	3 d Record	3–4 y n = 324	5590 kJ (1100) ~1336 kcal (277)	~31.7%E = 47 g (13)	~11.7%E = 17.3 g (5.3)	NR	NR	NR	NR	MUFA 16.5 g (5.1) PUFA 8 g (2.9)
			5–6 y n = 233	6370 kJ (1250) ~1522 kcal (298)	~31.9%E = 54 g (14)	~11.5%E = 19.5 g (6.2)	NR	NR	NR	NR	MUFA 18.8 g (5.3) PUFA 9.4 g (3.1)
Finland 2 children participating in DIPP study 2003–2005 Cross-sectional study	Kyttälä 2010 [76]	3 d Record	1 y n = 455	3800 (640) kJ ~908 kcal	29 (6)%E = 30 (8) g	11.6 (3.3)%E = 12 (4.2) g	NR	NR	NR	NR	MUFA 10.5%E = 10.5 g PUFA 5.1%E = 5.2 g
			2 y n = 230	4570 (930) kJ ~1092 kcal	30 (6)%E = 37 (12) g	13.2 (3.6)%E = 16.4 (5.9) g	NR	NR	NR	NR	MUFA10.2%E = 12.7 g PUFA 3.8%E = 4.8 g
			3 y n = 471	5210 (990) kJ ~1245 kcal	31 (6)%E = 43 (13) g	13.2 (3.1)%E = 18.7 (6.1) g	NR	NR	NR	NR	MUFA 10.5%E = 14.8 g PUFA 4.1%E = 5.7 g
			4 y n = 554	5650 (1050) kJ ~1350 kcal	31 (5)%E = 48 (13) g	13.7 (3.2)%E = 21.1 (6.8) g	NR	NR	NR	NR	MUFA13.7%E = 16.3 g PUFA 4.0%E = 6.2 g
			6 y n = 713	6350 (1170) kJ ~1518 kcal	31 (5)%E = 54 (15) g	13.7 (3.0)%E = 23.6 (7.3) g	NR	NR	NR	NR	MUFA 10.7%E = 18.5 g PUFA 4.2%E = 7.2 g
Finland 3 (Kuopio) PANIC Study 2007–2009 Cross-sectional study	Naveed 2020 [77]	4 d Record	6–9 y n = 487 (250 M, 237 F)	ALL: ~6799 kJ (1297) 1625 kcal (310) M: ~7150 kJ (1299.6) 1709 kcal (310.6) F: ~6430 kJ (1190) 1537 kcal (284.4)	ALL: 30%E = 54.6 g (14.9) M: 30.4%E = 57.8 g (14.5) F: 29.9%E = 51.1 g (14.5)	ALL: 12.2%E = 22.1 g (7.1) M: 12.3%E = 23.4 g (6.9) F: 12.1%E = 20.7(7.1)	ALL: 3.6%E 6.6 g (2.4) M: 3.6%E = 7.0 g (2.5) F: 3.6%E = 6.1 g (2.3)	ALL: 0.78%E = 1400 (500) mg M: 0.79%E; 1500 (500) mg F: 0.76%E; 1300 (400) mg	ALL: 100 (100) M: 100 (100) F:100 (100)	ALL: ~140 M: ~140 F: ~130	ALL: MUFA: 18.1 g (5.1) (10%) PUFA: 8.9 g (2.8) (4.9%E) ARA: 90 mg (100) EPA: 40 mg (100)
France 1 Nutri-Bébé study 2013 Cross-sectional study	Chouraqui 2020 [78]	3 d Record	12–17 Mon., n = 121	~3819.6 kJ (1054.8) 912.9 kcal (252.1) Median: ~3714 kJ 887.7 kcal	~28.1%E = 28.6 (10.7) g Median: ~27.4%E = 27 g	NR	~2.8%E; 2.844 (1.627) g Median: ~2.92%E 2.884 g	~0.42%E; 431 (250) Median: ~0.42%E; 417 mg	36 (68) Median: 18	NR	ARA: 16 mg (24) Median: 9 mg
			18–23 Mon., n = 120	~4241.3 kJ 1013.7 kcal (290.5) Median: ~4185.3 kJ 1000.3 kcal	~29.8%E; 33.6 (11.8) g Median: ~29.5%E; 32.8 g	NR	~2.74%E; 3.084 (1.638) g Median: ~2.83%E; 3.142 g	~0.38%E; 427 (227) Median: ~0.37%E; 416 mg	37(68) Median: 19	NR	ARA: 25 mg (42) Median: 16 mg
			24–29 Mon., n = 125	~4154.3 kJ (1214.6) 992.9 kcal (290.3) Median: ~4119.15 kJ 984.5 kcal	~32.6%E 33.7 (13.1) Median: ~29.6%E; 32.4 g	NR	~2.48%E; 2.559(1.779) g Median: ~2.10%E; 2.297 g	~0.34%E; 347 (215) mg Median: ~0.28%E; 304 mg	48(79) Median: 22	NR	ARA: 23 mg (25) Median: 19 mg
			30–35 Mon., n = 81	~4395.7 kJ (1928.4) 1050.6 kcal (460.9) Median: ~4267.3 kJ 1019.9 kcal	~30.6%E; 35.7 (20.8) Median: ~29.2%E; 33.1 g	NR	~2.17%E; 2.534 (1913) Median: ~2.1%E; 2.384 g	~0.31%E; 356 (289) mg Median: ~0.29%E; 327 mg	61(131) Median: 37	NR	ARA: 30 mg (35) Median: 24 mg
France 2 National (INCA 2) 2006–2007 Cross-sectional study	Guesnet 2019 [79]	7 d Record	3–5 y, n = 163	NR	38.4%E (5.1) = 62.1(16.9)	NR	3.7%E (2) 6.1 (4.2)	0.4%E (0.2) 700 (300)	107 (303)	184 (250)	ARA: 70 mg (70) EPA: 77 mg (215)
			6–8 y, n = 228	NR	38.2%E (4.2) 73.5 (19)	NR	3.5%E (1.3) 6.8 (2.9)	0.4%E (0.2) 800 (400)	80 (55)	144 (50)	ARA: 77 mg (38) EPA: 60 mg (45)
Germany 1 DONALD (Dortmund) 2010 Cross-sectional study	Libuda 2014 [80]	3 d Record	2–3 y 221 M, 206 F	Median: 4100 kJ ~980 kcal	Median: 34.6%E; 37.2 g	Median: 15.2%E; 15.9 g	NR	NR	NR	NR	Medians: MUFA: 15.7 g; 14.7%E PUFA: 5 g; 4.5 E
			4–12 y 296 M, 285 F	Median: 6500 kJ ~1554 kcal	Median: 33.8%E; 59.1 g	Median: 14.8%E; 25.9 g	NR	NR	NR	NR	Medians: MUFA: 23.7 g; 13.7%E PUFA: 8 g; 4.7%E
Germany 2 National KiGGS (EsKIMO module) 2006 Cross-sectional study	Stahl 2009 [81]	3 d Record	6–11 y n = 1234	Medians: M: 7580 kJ ~1812 kcal F: 7010 kJ ~1676 kcal	Medians: M: 32%E; 66 g F: 32%E; 60 g	Medians: M: ~14%E; 28 g F: ~ 14%E; 26 g	NR	NR	NR	NR	Medians: MUFA: ~11%E (M. 23 g; F: 20 g) PUFA: ~4%E (M. 8 g; F: 8 g)
Greece Crete, 2006–2007 Cross-sectional study	Smpokos 2014 [82]	3 d Record	5.7–7.6 y, n = 257	Mean (SE) M 8646 kJ (180) ~2066.4 kcal (43) F 7963 kJ (197) ~1903.2 kcal (47.08)	M 42.8 (0.5)%E F 43.1 (0.6)%E Medians M ~88.4 g F ~82.8 g	M 15.4 (0.3)%E F 15.3 (0.3)%E Medians M ~35.3 g F ~32.4 g	n-6 PUFA ^11^: M 8 g ~3.5%E F 7.4 g ~3.5%%E	n-3 PUFA ^14^: M 692 mg ~0.38%E F 590 mg ~0.28%E	NR	NR	MUFA: M 17.6%E (0.3) F 18.1%E (0.4) PUFA: M 4.7%E (0.1) F 4.7%E (0.1)
Ireland 1 National NCFS II 2019 Cross-sectional study	O’Connor 2021 [41]	4 d weighed Record + correction	5–8 y n = 300	~5773.9 kJ (979.1) 1380 kcal (234)	33.1(3.85)%E; 51.6 (11.0) g	14.2(2.17)%E; 22.1 (5.20) g	n-6 PUFA ^11^: 3.50 (0.59)%E; 5.45 (1.32) g	0.6 (0.16)%E 910 mg (270)	61.1 (46.6)	100.3	MUFA: 13.3%E (1.77) = 20.8 g (4.67) n-3 PUFA: 0.74 (0.18)%E; 1.15 (0.35) g PUFA: 5.49%E (0.97) = 8.51 g (2.09) EPA: 39.2 (29.2) mg
Ireland 2 National (NPNS) 2010–2011 Cross-sectional study	Walton 2017 [83]	4 d weighed Record + correction	1 y n = 126	4300 kJ (300) 1019 kcal (60)	33.8%E (1.3) 38.8 (2.9)	15.7%E (0.8) = 18.0 g (1.5)	NR	0.46 (0.04)%E = 530 (60) mg	30.3 (10)	64.6 (15.3)	MUFA: 11.7%E (0.5) = 13.5 g (1.1) n-3 PUFA: 0.52%E (0.05); 600 (100) mg PUFA: 4.1%E (0.3) = 4.7 g (0.5) EPA: 30.2 (7.8) mg
			2 y n = 124	4800 kJ (300) 1131 kcal (67)	32.8%E (1.3) 41.8 (3.2)	14.8%E (0.8) = 18.9 g (1.6)	NR	0.46 (0.05)%E = 580 (70) mg	40.2 (14)	68.2 (16.9)	MUFA: 11.1%E (0.6) = 14.2 g (1.2) n-3 PUFA: 0.52%E (0.05) = 700 (100) mg PUFA: 4.4%E (0.4) = 5.6 g (0.6) EPA: 28.6 (6.7) mg
			3 y n = 126	4800 kJ (300) 1151 kcal (69)	31.8%E (1.3) 41.3 g (3.2)	14.5%E (0.8) = 18.9 g (1.7)	NR	0.44 (0.04)%E 570 (70) mg	39.5 (14)	68.0 (17.1)	MUFA: 10.6%E (0.6) = 13.8 g (1.2) n-3 PUFA: 0.5%E (0.05) = 600 (100) mg PUFA: 4.3%E (0.4) = 5.5 g (0.6) EPA: 32.2 (9.1) mg
			4 y n = 124	5300 kJ (300) 1265 kcal (73)	31.5%E (1.3) 44.8 g (3.4)	14.1%E (0.8) = 20.1 g (1.7)	NR	0.44 (0.05)%E = 630 (80) mg	45.4 (15.4)	79.8 (19.9)	MUFA: 10.6%E (0.6) = 15.2 g (1.3) n-3 PUFA: 0.5%E (0.05) = 700 (100) mg PUFA: 4.3%E (0.4) = 6.1 g (0.7) EPA: 34.4 (9.3) mg
Italy National (INRAN-SCAI) 2005–2006 Cross-sectional study	Sette 2011 [84]	3 d Record	3–9.9 y n = 193	8000 kJ 1914 (488) kcal Median 8000 kJ 1906 kcal	37.4 (4.9)%E 79.5 (22.8) g Median 37.3%E 78.8 g	11.9 (2.5)%E 25.4 (8.5) g Median 11.7%E 24.6 g	NR	NR	NR	NR	MUFA: 17.4 (2.8)%E Median 4.4%E 37.0 (10.9) g Median 34.9 g PUFA: 4.5 (1.0)%E Median 4.4%E 9.8 (3.5) g Median 9.3 g
The Netherlands 1 2011–2014 Cross sectional study	Goldbohm 2016 [85]	2 d Record	1 y n = 411	4954 kJ (822) = ~1184 kcal (196)	28.7%E (4.8) 38 g (10)	10.3%E (1.9) 13.5 g (3.4)	5.1%E (1.7) = 6.7 g (2.7)	0.7%E (0.2) 900 (300)	20 (50)	~40	MUFA: 12.9(4.2) g = 9.8%E (2.4) n-6 PUFA: 5.7 (1.7)%E = 6.8 (2.7) g n-3 PUFA: 1.0 (0.3) g = 0.7%E (0.2) PUFA: 8.0 (3.1) g = 6.1%E (1.9) EPA: 20 mg (30)
			2 y n = 497	5424 kJ (872) = ~1296 kcal (208)	28.9%E (4.5) 42 g (10)	10.3%E (1.8) = 14.8 g (3.7)	5.0%E (1.5) = 7.2 g (2.6)	0.7%E (0.2) 1000 (300)	20 (40)	~30	MUFA: 9.9%E (2.3) 14.4 g (4.5) n-6 PUFA: 5.0 (1.5)%E 7.3 (2.6) g n-3 PUFA: 0.7%E (0.2) 1.0 g (0.4) PUFA: 5.9%E (1.7) 8.6 g (3.0) EPA 10 mg (20)
			3 y (3 y–48 Mon.) n = 410	5841 kJ (923) = ~1396 kcal (221)	29.3%E (4.3) 45 g (11)	10.4%E (1.7) = 16.1 g (3.8)	5.1%E (1.5) = 7.9 g (3.0)	0.7%E (0.2) 1000 (400)	20 (40)	~30	MUFA 10.2%E (2.2) 15.8 g (4.4) n-6 PUFA 5.1 (1.5)%E 8.0 (3.0) g n-3 PUFA 0.7%E (0.2) 1.1 g (0.4) PUFA 6.0%E (1.7) 9.4 g (3.4) EPA 10 mg (30)
The Netherlands 2 National Survey 2012–2016 Cross sectional study	Van Rossum 2020; Steenbergen 2021 [35,86]	2x 24 h + FFQ	1–3 y M: n = 332 F: n = 340	M: 5500 kJ ~1315 kcal Median 5300 kJ ~1267 kcal F: 5200 kJ ~1243 kcal Median 5100 kJ ~1219 kcal	M: 29.5%E = 44 g Median 29.4%E = 42 g F: 29.4%E = 42 g Median 29.4%E = 41 g	M: 11.1%E = 16 g Median 11%E = 16 g F: 11%E = 16 g Median 10.9%E = 16 g	M: 4.6%E = 7 g Median 4.5%E = 7 g F: 4.6%E = 7 g Median 4.5%E = 6 g	M: 0.6%E = 900 mg Median 0.6%E = 800 mg F: 0.6%E = 900 mg Median 0.6%E = 800 mg	NR	M: 51 Median 34 F: 63 Median 43	Cis- UFA FA M: 15.8%E = 23 g Median 15.6%E = 22 g F: 15.6%E = 23 g Median 15.4%E = 22 g
			4–8 y M: n = 261 F: n = 259	M: 7600 kJ ~1816 kcal Median 7400 kJ ~1625 kcal F: 6800 kJ ~1625 kcal; Median 6700 kJ ~1601 kcal	M: 31.7%E = 65 g Median 31.6%E = 63 g F: 31.8%E = 58 g Median 31.7%E = 57 g	M: 11.8%E = 24 g Median 11.7%E = 24 g F: 11.8%E = 22 g Median 11.7%E = 21 g	M: 5%E = 11 g Median 4.9%E = 8 g F: 5.1%E = 10 g Median 5.0%E = 7 g	M: 0.6%E = 1300 mg Median 0.6%E = 1200 mg F: 0.6%E = 1100 mg Median 0.6%E = 1100 mg	NR	M: 74 Median 50 F: 81 Median 56	Cis- UFA FA M: 17%E = 36 g Median 16.8%E = 34 g F: 17.2%E = 32 g Median 17%E = 31 g
Poland One medium size city & his area Date of data collection not found Cross-sectional study	Merkiel 2014 [87]	3 d Record	6 y N = 120	7707 kJ (1335) 1840 kcal (319) Median (SE) 7589 kJ (122) 1812 kcal (29)	32.7 (4.0)%E = 68.2 (16.3) g Median 32.7 (0.4)%E = 66.1 (1.5) g	14.5 (2.3)%E = 29.79 (8.21) g Median 14.4 (0.2)%E = 29.13 (0.75) g	NR	NR	NR	NR	MUFA: 12.3 (1.8)%E; 25.36 (6.41) g Median 12.2 (0.2)%E 24.38 0.58 PUFA: 4.1 (1.4)%E = 8.26 (2.87) Medians: 3.8 (0.1)%E = 7.87 (0.26) g
Spain 1 National (ENALIA) 2013–2014 Cross-sectional study	López-Sobaler 2019 [88]	2 d Record + FFQ	1–3 y n = 407 M 218 F 189	M: ~6188.1 kJ (895); 1479 kcal (214) F: ~5773.9 kJ (723.8); 1380 kcal (173) Medians: M: ~6163 kJ; 1473 kcal F: ~5740 kJ; 1372 kcal	M: 34.6 (3.4)%E; 57.7 g (11.9) F: 34.6 (3.1)%E; 52.8 g (8.7) Medians: M: 34.6%E; 57.1 g F: 34.6%E; 52.2 g	M: 11.9 (3.2)%E; 19.8 g (7) F: 11.9 (3.6)%E; 18.3 g (6.5) Medians: M: 12%E; 19.5 g F: 12%E; 17.7 g	NR	NR	NR	NR	MUFA M: 11.3%E; 19.2 (6.1) g F: 11.6%E; 17.8 (5.0) g Medians: M 11:.3%E; 18.8 g F: 11.5%E; 17.5 g PUFA Means: M: 4.9 (0.9)%E; 8.4; (2.2) g F: 5.0 (0.7)%E; 7.7 (1.3) g Medians: M: 4.8%E; 8.1 g F: 4.9%E; 7.5 g
			4–8 y n = 418 M 211 F 207	M ~ 7727.8 kJ (882) 1847 kcal (211) F ~6912 kJ (657) 1652 kcal (157) Medians M ~7694.4 kJ 1839 kcal F ~6887 kJ 1646 kcal	M 34.8 (2.9)%E = 71.9 g (8.7) g F 35.6 (2.8)%E = 65.4 g (7.2) g Medians M 34.8%E = 71.5 g F 35.6%E = 65.1 g	M 13.1 (1.6)%E = 27.1 g (4.6) F 13.1 (1.9)%E = 24 g (4) Medians M 13.1%E = 26.8 g F 13.1%E = 23.7 g	NR	NR	NR	NR	MUFA M 13.2%E = 27.4 g (4.3) F 14.1%E = 25.9 g (2.6) Medians: M 13.2%E = 27.1 g F 14%E = 25.8 g PUFA M 5.1%E = 10.5 g (0.8) F 4.9%E = 9.1 g (1.7) Medians: M 5.1%E = 10.5 g F 4.8%E = 8.9 g
Spain 2 Urban areas across 9 Spain geographical areas (EsNuPI Study 2018–2019) prospective, cross-sectional, observational	Madrigal 2020 [89,90]	2x 24 h + correction	1–<3 y n = 162	~5142 kJ (1452) 1229 kcal (347) Median ~5083.6 kJ 1215 kcal	36.7%E; 49.6 g (16.84) Median 47.34 g	13.1%E = 17.58 g (7.81) Median 16.88 g	~3.7% = 5.03 g (3.28) Median 4.45 g	~0.26%E = 360 (150) mg Median 350 mg	80 (120) Median 20	~130	MUFA: 19.19 g (8.75) 14.5%E Median 19.25 n-6 PUFA: 5.09 g (3.28) = 3.6%E Median 4.51 g n-3 PUFA: 0.53 g (0.28) 0.4%E Median 0.49 g PUFA: 5.66 g (3.79) 3.9%E Median 5.15 g DPA: 30 mg (30) Median 20 EPA: 50 mg (70) Median 10 ARA: 60 mg (50) Median 50
			3–<6 y n = 244	~6242.5 kJ (1451.8) 1492 kcal (347) Median ~6263.4 kJ 1497 kcal	36.8%E; 60.83 (17.89) g Median 60.34 g	13.5%E = 22.43 (7.20) g Median 22.18 g	~3.8%E; 6.36 (3.24) g Median 5.85 g	~0.27%E 450 (160) mg Median 430 mg	80 (140) Median 20	~130	MUFA: 25.56 g (8.21) 15.5%E Median 25.29 g n-6 PUFA: 6.44 g (3.25) 3.9%E Median 5.91 g n-3 PUFA: 0.63 g (0.27) 0.4%E Median 0.59 g PUFA: 7.72 g (3.41) 4.2%E Median 7.16 g DPA: 50 mg (40) Median 40 EPA: 50 mg (70) Median 10 ARA: 80 mg (50) Median 70
Sweden National Food Survey 2003	Enghardt Barbieri, 2003 [91]	4 d Record	4 y n = 590	6300 kJ 1505 kcal	31.7%E (4.4) = 54 g (14) Median: 31.7%E = 53 g	14.4%E (2.5) = 25 g (7) Median: 14.3%E = 24 g	3%E = 5 g (1.6) Median 4.8 g	0.6%E = 1000 (300)mg Median 900 mg	100 (180) Median 60	140	MUFA: 11.3%E (1.8) = 19 g (5) Medians: 11.2%E = 19 g n-6 (LA+ARA): 3%E (0.8); 5 (1.7) g Medians: 2.8%E = 4.9 g n-3 (ALA+ EPA+ DPA+ DHA): 0.7%E (0.2); 1.1(0.5) Medians: 0.6%E = 1.1 g PUFA: 3.7%E (0.9–1.0) = 6 g (2) Medians: 3.5%E = 6 g EPA 40: (80)mg Median: 10 mg DPA: 30 (50) mg Median: 20 mg
Turkey Nutrition and Health Survey 2010 Nationally representative Cross-sectional survey	Reported in Rippin 2018 (review) [14]	24 h FFQ, or face to face interview	2–5 y	M 5500 kJ 1253 kcal; F 5300 kJ 1190 kcal	M ~37.4%E = 52 g; F~37.0%E = 49 g	M ~12.9%E = 17.9 g; F ~12.8%E = 16.9 g	n-6 PUFA ^11^: M ~9.1%E = 12.6 g F ~8.7%E = 11.5 g	n-3 PUFA ^14^: M: ~0.7%E = 1000 mg F 0.7%E = 900 mg	NR	NR	NR
			6–8 y	M 1587; F 1510	M: ~35.2%E = 62 g; F: ~35.8%E = 60 g	M ~12.0%E = 21.1 g; F ~11.7%E = 19.6 g	n-6 PUFA ^11^: M ~8.8%E = 15.6 g F ~9.3%E = 15.6 g	n-3 PUFA ^14^: M ~0.6%E = 1100 mg; F ~0.7%E = 1200 mg	NR	NR	NR
UK 1 National (NDNS) 2008–2011 Cross-sectional survey	Gibson 2014 [92]	4 d Record	1.5–3 y n = 185	4700 kJ 1110 kcal	34.1%E = 42 g	15.1%E = 18.8 g	n-6 PUFA ^11^: 3.8%E = 4.7 g	n-3 PUFA ^14^: 0.7%E = 800 mg	NR	NR	MUFA: 13.9 g (11.2%E)
UK 2 National (DNSIYC) 2011 Cross-sectional survey	Gibson 2014 [92]	4 d Record	12–18 Mon. n = 1275	4100 kJ 967 kcal	35.4%E = 38 g	16.3%E = 17.5 g	n-6 PUFA ^11^: 3.7%E = 4 g	n-3 PUFA ^14^: 0.7%E = 700 mg	NR	NR	MUFA: 12.4 g (11.5%E)
UK 3 National (NDNS) 2014–2016 Cross-sectional survey	NDNS website, results from y 7–8 of the Rolling Programme [93]	4 d Record	1.5–3 y n = 250	~4502 kJ (1013) 1076 kcal (242) Median 4309.5 kJ 1030 kcal	34.4%E (5.4) = 41.3 (12)g Median: 34.6%E = 39 g	14.5%E (3.4) = 17.5 (6.2)g Median: 14.7%E = 16.6 g	NR	NR	NR	NR	NR
			4–10 y n = 514	5991.5 kJ (1368.2) 1432 kcal (327) Median: 4309.5 kJ 1400 kcal	33.4%E (4.4) = 53.5 g (15.6) Median: 33.7%E = 51.7 g	13 (2.7)%E = 20.9 (7.1) g Median: 12.9%E = 19.9 g	NR	NR	NR	NR	NR
UK 4 National (NDNS) 2008–2009 & 2011–2012 Cross-sectional survey	SACN Report 2019 [24]	3–4 d Record	4–10 y n = not indicated	NR	NR	NR	n-6 PUFA ^11^: 4.4 (1.3)%E 7.5 (2.9) g Median: 4.2%E = 7 g	n-3 PUFA ^14^: = 0.8 (0.3)%E 1400 (600) mg Median: 0.8%E = 1300 mg	NR	NR	MUFA: 20.5 (6.3) g = 12 (2.1)%E Median: 20 g = 11.9%E
Middle East											
Lebanon National 2012 Cross-sectional study	Reported in Nasreddine 2018 (review) [16]	24 h	2–5 y n = 531; M: n = 284; F: n = 247	~6618.25 kJ (2689.1) 1581.8 kcal (642.7)	38.8%E = 69.9 g (34.7)	12.7%E	NR	NR	NR	NR	NR
United Arab Emirates (UAE) National Cross-sectional study	Ali 2013 [94]	24 h	6–8 y M: n = 78 F: n = 85	M: ~6389 kJ (SE 284.1) 1527 kcal (SE 67.9) F: ~6125.4 kJ (SE 261.9) 1464 kcal (SE 62.6)	M: 26.3%E (SE 0.9) F: 24.4%E (SE 1.5)	M: 9.77%E (SE 0.6) F:8.9%E (SE 0.7)	NR	NR	NR	NR	NR
Oceania											
Australia 1 Melbourne 2008–2010 Control arm of a RCT	Lioret 2013 [95]	3x 24 h	18 Mon. n = 177	4473.3 kJ (779.4) 1069 kcal (186.3) Median 4408.4 kJ 1054 kcal	~32.75%E = 38.9 g (9.3) Median ~32.5%E = 38.1 g	~16.7%E = 19.8 g (5.2) Median ~16.6%E = 19.4 g	NR	NR	NR	NR	NR
Australia 2 National (ANCNPA) 2007 Cross-sectional survey	Meyer 2011 [36]	2x 24 h	2–3 y n = 1071	6038 kJ ^15^ 1443 kcal	NR	NR	~3.3%E = 5.3 g (2.7) Median 4.7 g	~0.53%E = 850 mg (510) Median 750 mg	NR ^16^	NR ^16^	
			4–8 y n = 1216	7245 kj ^15^ 1732 kcal	NR	NR	~3.5%E = 6.7 g (3.4) Median 6.0 g	~0.57%E = 1010 mg (560) Median 910 mg	NR ^16^	NR ^16^	
Australia 3 National (ANCNPA) 2007 Cross-sectional survey	Rahmawaty 2013 [37]	2x 24 h	2–3 y n = 1071	NR	NR	NR	NR	NR	31.2 (77.2) Median 3.9	~47.7 Median ~9.2 mg	LC n-3 PUFA: (EPA+DPA+DHA) 59.7 (128.5) Median: 21.5 mg DPA: 12 mg (18.6) Median: DPA 6.2 mg EPA 16.5 mg (40.5) Median: 5.3 mg
			4–8 y n = 1216	NR	NR	NR	NR	NR	35.9 (92.7) Median 5.1	55.1	LC n-3 PUFA (EPA+DPA+DHA): 70.5 (152.7) Median: 26.1 DPA: 15.3 (23.5) Median: 8.2 mg EPA: 19.2 (47.6) Median: 6.7 mg
Australia 4 National (ANCNPA) 2007 Cross-sectional survey	Rangan 2014 [38]	24 h	2–8 y n = 2078 (only plausible energy intake reporters)	7010 kJ (40) ~1675 kcal (9.56)	30.4%E (0.15)	13.8%E (0.09)	NR	NR	NR	NR	MUFA: 10.4%E (0.07) PUFA: 3.8%E (0.04)
Australia 5 Adelaide (South) 2005–2007 Cross-sectional survey	Zhou 2012 [96]	3 d Record	1–2 y n = 92	Median: 4241 kJ 1013 kcal	Median: 40 g ~35.5%E	Median: 20 g ~17.7%E	Median: n-6 PUFA ^11^ ~2.11%E 2.380 g	Median: n-3 PUFA ^14^ 474 mg ~0.42%E	NR	NR	Median: n-3 LCPUFA (EPA + DPA + DHA): 22 mg
			>2–3 y n = 67	Median: 5024 kJ 1201 kcal	Median: 46 g ~34.5%E	Median: 23 g ~17.2%E	Median: n-6 PUFA ^11^ 3.123 g ~2.34%E	Median: n-3 PUFA ^14^ 562 mg ~0.42%E	NR	NR	Median: n-3 LCPUFA (EPA + DPA + DHA): 20 mg
			>3–4 y n = 70	Median: 5049 kJ 1207 kcal	Median: 43 g ~32.1%E	Median: 20 g ~14.9%E	Median: n-6 PUFA ^11^ 3.478 g ~2.6%E	Median: n-3 PUFA ^14^ 459 mg ~0.34%E	NR	NR	Median: n-3 LCPUFA (EPA + DPA + DHA): 26 mg
			>4–5 y n = 68	Median: 5982 kJ 1430 kcal	Median: 51 g ~32.1%E	Median: 24 g ~15.1%E	Median: n-6 PUFA ^11^ 4.357 g ~2.7%E	Median: n-3 PUFA ^14^ 581 mg ~0.37%E	NR	NR	Median: n-3 LCPUFA (EPA + DPA + DHA): 34 mg

Abbreviations: 24 h: Single administration of 24 h dietary recall on whole sample; 24 h × (24 h): Single administration of 24 h dietary recall on whole sample plus repeat(s) on subsample; %E: % of total daily energy intake; ALA: alpha-linolenic acid; DHA: docosahexaenoic acid; DPA: docosapentaenoic acid, 22:5 n-3; EPA: eicosapentaenoic acid; F: females; FA: fatty acid; FFQ: food frequency questionnaire; LA: linoleic acid; LC-PUFA: long chain polyunsaturated fatty acids; M: males; Mon.: month; MUFA: monounsaturated fatty acids; PUFA: polyunsaturated fatty acids; SFA: saturated fatty acids; UFA: unsaturated fatty acids; WFR: weighed food record. y: year. STUDIES: ANCNPA: Australian National Children’s Nutrition and Physical Activity Survey; CCHS: Canadian Community Health Survey; DAGIS: Increased Health and Wellbeing in Preschools (Finland); DIPP: Type 1 Diabetes Prediction and Prevention study (Finland); DONALD: Dortmund Nutritional and Anthropometric Longitudinally Designed Study (Germany); DONGuRI: Dietary Observation and Nutrient intake for Good health Research in Japanese young children; ENALIA: National Dietary Survey on the Child and Adolescent Population project in Spain; ENSANUT: Encuesta Nacional de Salud y Nutrición (Mexico); EsKiMo: Eating study as a KiGGS Module (Germany); EsNuPI: Nutritional Study in the Spanish Pediatric Population; FECHIC: Food Environment Chilean Cohort; FITS: Feeding Infants and Toddlers Study; GUSTO: Growing-up in Singapore (mother-offspring cohort study); INCA: Étude Individuelle Nationale des Consommations Alimentaires; INRAN-SCAI: Italian National Food Consumption Survey; KiGGS: German Health Interview and Examination Survey for Children and Adolescents; KNHANES VI: sixth Korea National Health and Nutrition Examination Survey; NCFS: Irish National Children Food Survey; NDNS: National Dietary Nutrition Survey (UK); NHANES: National Health and Nutrition Examination Survey (US); NHNSJ: National Health and Nutrition Survey (Japan); NNS: National Nutrition Survey (Australia); NPNS: National Pre-School Nutrition Survey (Ireland); PANIC: Physical Activity and Nutrition in Children; PDIS: Provincial Dietary Intake Study (South Africa; Follow-up of the 1999 National Food Consumption Study); RISKESDAS: Indonesia Basic Health Research. Energy intakes, expressed in Joules, were converted to kcal, or vice versa, using the factor 1 MJ = 239.006 kcal, and both units can be found in Table 2. Except for LC n-3 PUFA, when fat and fatty acid intakes were given in absolute amounts, the intake value was calculated in %E using the total daily energy intake found in the same paper or another publication of the same population using a factor of 9 kcal/g fat or fatty acid. ^1^ All along the table, “~ kcal” or ”~kJ” means that we converted the energy unit (1 kJ = 0.239006 kcal). ^2^ All along the table, “~x%E” means that we calculated the %E for fat (or FA) (~x%E = 100 × daily mean (or median) fat (or FA) intake (in g/day) × 9/total daily mean (or median) energy intake (in kcal). ^3^ The SE were calculated using the reported 95th confidence interval (95% CI = mean ± [1.96 * SE]), all along the tables, for papers that reported the 95% CI. ^4^ Energy intake values for this study were found in Garriguet, D. [97] ^5^ The mean of EPA + DHA was not reported while mean EPA and mean DHA were available. All along the table, “~EPA + DHA” means that we calculated the sum based on the average of the two individual FA. ^6^ Calculated based on daily energy intake of 1141 kcal/day as reported in Butte et al., [29] ^7^ Calculated based on daily energy intake of 1308 kcal/day as reported in Butte et al., [29] ^8^ Calculated based on daily energy intake of 1534 kcal/day at 4–5 y as reported in Hamner et al., [98] ^9^ Calculated based on daily energy intake of 1214 kcal (at 1 year) to 1534 kcal at 4 years as reported in Hamner et al., [98] ^10^ Calculated based on daily energy intake of 1961 kcal (for 6–11 years-old children) as reported in Murakami & Livingstone [99] ^11^ In this study (as in 10 other included studies) n-6 PUFA was assessed while LA was not; we reported n-6 PUFA value in the “LA” column; indeed, LA intake constitutes ~97% of n-6 PUFA intake [54,73]. Since both values are very close, we used the value for “n-6 PUFA” as estimation for “LA”. ^12^ In this study (as in 3 studies from Japan), n-3 PUFA was assessed while ALA was not. Given the high EPA and DHA intake in Japan, we estimated ALA intake as 60% n-3 PUFA intake, as per (Kakutani et al., [100] and Kusumoto et al., [101]). ^13^ Energy intake values for this study were calculated from energy intake values from the National Japanese studies: we calculated the average of 2005–2008 studies for males and females separately. ^14^ In this study (as in 7 other studies) n-3 PUFA was assessed while ALA was not and we reported n-3 PUFA value in the “ALA” column; ALA intake constitutes ~87–90% of n-3 PUFA intake [Ramirez Silva (54], Kim [73]). Since both values were very close, we used the value for “n-3 PUFA” as estimation for “ALA”. ^15^ Energy intake values for Australia ANCNPA were found in [102] ^16^ Long chain n-3 PUFA (EPA, DPA and DHA) in the paper of Meyer 2011 were not considered because they were recalculated and reported in Rahmawaty [37] after identification of errors in LC n-3 PUFA composition of certain foods (e.g., margarines) in the AUSNUT database used for the paper of Meyer [36].

#### 3.2.2. Recommended Cut-Off Values Used as a Basis of Comparison between Effective and Recommended Intake

##### Total Fat

Observed intakes of total fat were compared with the recommended intake using the highest value recommended for the age group. Indeed, the recommendations are to have a gradual reduction in the fat contribution to the diet between the ages of 6 months and 3 or 4 years [1,18]. Infants and children fed human milk (as recommended by the FAO/WHO until the age of 2 years [103]) consume 50%E as fat, while the recommendations for fat between 2 and 18 years are 25–35%E [17]. Thus, taking the upper end of the range, it was decided that the fat adequacy for our assessment would be of minimum 35%E in children 1–3 years (according to EFSA) and between 25 and 35%E in children 3–7 years (according to FAO/WHO).

##### Total SFA

Three thresholds were used for the comparison of children’s observed intake with the recommended intake, and these were according to the current 8% limit of FAO/WHO [17] and the 10% limit proposed by FAO/WHO in the latest draft guidelines on saturated fatty acids and trans-fatty acids for adults and children [21]: (1) countries with observed intakes lower than 8%, (2) countries with observed intake between 8 and 10%, (3) countries with observed intakes higher than 10%. Because there is no SFA intake limitation for children less than 2 years, no comparison was made for SFA for this age group.

##### LA

EFSA AI is at least 4%E, higher than that of the FAO/WHO, because it is mainly based on current intakes in the European population, while lower amounts are still adequate for the prevention of deficiency symptoms [79]. Thus, we used three thresholds for the comparisons between LA observed and recommended intakes: (1) countries with observed intakes lower than 3% (for 1–2 year old children) or lower than 2% (for 2–7 years old children), which correspond to the lower AI values from FAO/WHO for these 2 age groups; (2) countries with intake between 3 and 4% (for 1–2 years old children) or 2 to 4% (for 2–7 years old children), which correspond to the range between the FAO/WHO lower recommended AI value and the EFSA minimum AI; (3) countries with LA intakes higher than 4% (EFSA minimum AI).

##### ALA

The AI for ALA is similar across ages and for the two authorities (FAO/WHO and EFSA), thus studies were sorted into two groups: those where the mean intake was lower than 0.5%E and those where it was equal to or higher than 0.5%E.

##### EPA and DHA

FAO/WHO recommendations for EPA and DHA are age-specific, while those of EFSA are not. The intakes of EPA and DHA were compared with FAO/WHO recommendations (using the lower recommended value of the range as a reference for each of our age groups). Minimum reference intakes were: 100 mg (DHA) for children aged 1–2 years, 100 mg EPA+ DHA for those aged 2–3 years, 125 mg for those aged 3–5 years and 175 mg for those aged 5 to 7 years. Intakes were also compared against the EFSA recommendation (250 mg/day after 2 years), which is much higher than that of the FAO/WHO. Table 3 summarizes the cut-off values used for our assessment.

#### 3.2.3. Recommended Intake vs. Effective Intake: Comparisons by Age Group

Supplementary Tables S4–S7 give the numbers extracted from Table 2 and sorted for each of our four age groups (Table S4: intakes in children 1–2 years; Table S5: intakes in children 2–3 years; Table S6: intakes in children 3–5 years; Table S7: intakes in children 5–7 years) that were used for the assessment vs. the authority recommendations. Graphical representations of these intake data for total fat, total SFA, LA, ALA and n-3 LC-PUFA can be found in the Supplementary Figures S1–S5. The results of the assessment vs. the recommendations are given in Table 3, as the proportion of studies where the data were not in line with recommendations for each of the fatty acids and each of the four age groups.

##### Children 1–2 Years

The intake of total fat was below the lower recommendation in 23 of 26 (88%) studies (range 23 to 36.7%E). LA intake was below the FAO/WHO recommendation of 3%E in 24% of studies and below EFSA AI of <4%E in 59% of studies. ALA intake was below the AI of 0.5%E in 42% of studies. Daily DHA intake ranged between 19.5 to 120 mg and was below the recommendation of 100 mg per day in all of the recovered studies that assessed DHA intake except in one study done in Singapore [69]. Among this age group, two studies, one in China [61] and one in France [78] reported intakes of total fat, LA, ALA and DHA which were all outside (below) the recommendations.

##### Children 2–3 Years

Total fat intake ranged between 15 and 38.8%E and was below 35%E in 89% of studies. Intake of SFA had a wide range, from 3.6% to 17.2%E, and was above the recommendation of 10%E in 73% of the studies, notably in all the studies from North America, Europe, and Oceania. SFA intake was less than 8%E in only two studies from The Gambia [43] and Bangladesh [60], two countries with very low fat consumption (15%E and 17.2%E, respectively). LA intake was within the FAO/WHO recommendation for all studies and below EFSA AI in 44% of studies. Surprisingly, data from the National Study in Turkey show an exceptionally high LA intake of 9.1%E [14]. ALA intake was below the recommendation in 50% of studies. Daily EPA + DHA intake ranged from 24.7 to 152 mg and was below the FAO/WHO recommendation of 100 mg/day in 7 of the 10 studies for which these data were recovered, except two local studies done in Vancouver (Coastal area in Canada) [49,52] and one subnational study done in urban areas in Spain [89] with daily intakes of 100, 152 and 130 mg, respectively. EPA + DHA consumption was below the EFSA recommendation (250 mg/day) in all studies. Among this age group, two studies, one in Canada [48] and one in Ireland [83] reported intakes which were all outside of the recommendations: intakes of total fat, ALA and DHA were below and saturated fat was above the recommendations, while LA was not reported.

##### Children 3–5 Years

Total fat intake ranged from 12.9 to 38.8%E. It was below 25%E in 16% of studies and above 35%E in 17% of studies. Total SFA intake ranged from 3.4% to 14.9%E and was above the recommendation of 10%E in 69% of the studies, notably in all studies done in Europe and Oceania. Total SFA intake was less than 8%E in only two studies, one in South Africa [44] (sub-group aged 4–5.9 years from one settlement) and one in Bangladesh [60], two populations where fat consumption was low (23.8%E and 12.9%E, respectively). LA intake was within the FAO recommendation of 2%E in all studies and below the EFSA AI in 41% of studies. Data from the National Study in Turkey show an exceptionally high LA intake of 9.1%E [14]. ALA intake was below the recommendation in 42% of the studies. EPA + DHA intake ranged from 24.7 to 184 mg and was below the FAO/WHO recommendation of 125 mg/day in 9 of the 14 studies done in 11 countries for which these data were recovered. Studies with an intake >125 mg/day were two local studies (one in Vancouver (Coastal area in Canada) [49]; one in Japan [63], one sub-national study in urban areas in Spain) [89] and two national studies (France [79] and Sweden [91] with daily intakes ranging from 130 to 184 mg/day. EPA + DHA intake was below the EFSA recommendations of 250 mg/day in all studies.

##### Children 5–7 Years

Total fat intake ranged between 17.8 and 42.8%E. It was below 25%E in 9% of studies and above the upper range of the recommendation in 19% of studies. Intake of SFA ranged from 7.9% to 15.4%E and was above the 10%E recommendation in 73% of the studies, notably in all of the studies done in Europe and in Oceania. Total SFA intake was less than 8%E in only a population sub-group (aged 4–5.9 years from one settlement) in a study from South Africa [44], where fat consumption was low (23.8%E). LA intake was within the FAO/WHO recommendation for all studies and below the EFSA AI in 50% of studies. As per the other age groups, data from the National Study in Turkey showed an exceptionally high LA intake of 9.8%E [14]. ALA intake was below the AI in 42% of studies. EPA + DHA intake ranged from 30 to 144 mg and was below the FAO/WHO recommendation of 175 mg per day in all 11 studies for which DHA data was recovered for 5–7 years old children, done in 10 countries. Among this age group, three studies in Europe reported intakes which did not meet the recommendation for total and saturated fat (too high), and ALA and DHA (too low) [79,82,89], despite having intakes of LA within 2–4% of energy.

## 4. Discussion

### 4.1. Summary of Findings

This review provides an overview of the available FAO/WHO and EFSA recommendations for total fat and specific fatty acids and the dietary intakes of children worldwide aged 1–7 years are compared with these recommendations. The 65 studies identified, conducted in 33 countries, show that total fat intake was generally lower than recommended in 1–3 year-old children (88% of studies) and total SFA intake higher than the limit of 10%E in about 70% of children aged 2–7 years. LA intake was below FAO/WHO recommendation (<3E%) in 24% of studies in younger children (1–2 y) and adequate in older children (2–7 years) when assessed against FAO/WHO, while it was lower than EFSA AI in about 50% of studies. ALA intake was below the AI in almost half of the recovered studies and DHA [or EPA+DHA] intake was lower than the FAO/WHO recommendations in most studies and lower than EFSA recommendations in all studies of children more than 2 years.

### 4.2. Methodological Considerations

Data were recovered from many geographic regions, but there is limited information from Africa, South America, and China and we did not find any data from India, the country with the highest number of young children [104]. We did not perform a thorough comparison between countries because the studies differed in their objectives and dietary assessment methodologies. It is well known that all dietary intake assessment methods have their own limitations. Dietary recalls typically rely on memory, while dietary records may potentially be affected by reactivity, but both, at least when weighed records are used and several days are recorded, have the advantage of capturing more detail than food frequency questionnaires [105]. However, both recalls or records may underestimate the intake of food items which are consumed less frequently than others. This is important for FA for which one food item may represent >50% of its intake, such as EPA and DHA, for which fish is the major food source [106]. The method of choice for EPA + DHA intake would be a combination of recalls or records with a validated food frequency questionnaire since in most countries, fish/seafood are typically not consumed very frequently (e.g., daily) [106]. The importance of measurement method is nicely illustrated by the intra-country variability observed in Canadian children for the ALA intake (twice as high in the study of Madden et al., [51] vs. that of Lien et al., [50], which is likely related to methodological differences (chemical analysis of 3 days food collection aliquots vs. parental food records) rather than a real difference in intake per se.

The food databases used to calculate FA intake were not of the same quality, with some, (e.g., the US, French, Australian, and Indonesian databases) being more complete than others with respect to the FA composition of foods. In some national databases, the level of PUFA, in particular, DHA, were not available or were available only for a limited number of foods (e.g., Chinese database), thus, intake may be underestimated in some studies. Also, and partly because of this, only 23 studies reported DHA intake.

Most studies presented results as mean values, although several studies report median. The medians may be more appropriate for some FA such as DHA or the sum of EPA+ DHA, where the intake is not well distributed among the population. Nevertheless, regardless of the variable (mean or median) used, we found that DHA intake was very low.

### 4.3. Contribution of Breastmilk to Fat and Fatty Acid Intake

Thirteen studies among the 1–2 year-old age group included breastmilk intake in their assessment (Table S3). For those studies, intake of total fat and PUFA was still lower than the recommendations. Human milk is a source of LC-PUFA, the levels of which are dependent on multiple maternal factors including diet [107]. Given that in these studies the LC-PUFA content of the milk was not measured and information on maternal factors was not reported, it is difficult to assess the contribution of breastmilk to LC-PUFA intake in this age group. Of the thirteen studies that reported breastmilk intake, two of these studies from Africa discussed the contribution of breastmilk to fat and energy intakes. Prentice et al., [43] reported that breastmilk contributed significantly to fat intake up to 17 months (50%E), which declined thereafter to 30% and 15% at 17 and 24 months, respectively. In a more recent study from South Africa, Steyn et al., [45] reported that breastmilk contributed to 18.7% of total energy intake up to 3 years. Thus, in infancy and early childhood, breastmilk intake can contribute significantly to intake of energy and fat. Therefore, it is possible that the studies that did not include breastmilk intake in their assessment (n = 6) or that did not specify whether breastmilk was included or not (n = 12), underestimated intake of total fat and LC-PUFA.

### 4.4. Total Fat

While total fat intake is too low in 88–89% of studies in the younger age groups (1–3 years), for the older age groups, both low- (poor rural areas in Africa or Asia, or National surveys from South Korea, the Philippines or United Arab Emirates [esp. girls]) and high- (esp. in Mediterranean countries) fat intakes were reported. These results differ from those of previous reviews [14,15], which concluded that total fat intake in children less than 10 years of age was too high in most countries. This difference is because of the large age range used in these studies and the 30%E maximum RNI used (as per 2003 WHO adult recommendation [108]). This relatively low value of 30%E may not be an appropriate comparison to use for children of a young age. The high fat consumption observed in 3–7-year-old children in several Mediterranean countries is in accordance with the findings of Nasreddine [16] in school age children of the Eastern Mediterranean region.

Providing children with adequate dietary fat is of great importance given the essentiality of dietary fat and specific fatty acids for healthy growth and development in early life [1]. Dietary fat is energy dense and thus helps children meet the high energy needs of rapid growth, without ingesting large volumes of food. Given the rising prevalence of childhood obesity, and its high energy density, dietary fat is greatly scrutinized and suspected as a causal factor. Results of a recent Cochrane review commissioned by the WHO suggest that in trials among children aged 2–18 years where a lower fat intake (30%E or less) was provided compared with usual or modified fat intake, small reductions in body mass index, total- and LDL-cholesterol were observed [109]. However, in two of the three identified intervention studies, children were hypercholesterolemic, which limits the applicability of the findings to the general population of healthy children.

In the same review of Naude et al., [109], half of the 21 included cohort studies suggested that increased total fat intake was associated with increased body fatness (body weight and body mass index), nevertheless, some studies showed the opposite, and given the general low quality of recovered studies, no conclusion could be reached. It should be noted that twenty-three of the 24 included studies in Naude et al.’s review were done in children from high income countries. Moreover, given the design of the review (only randomised controlled trials included), some relevant studies were not included, such as the long-term follow-up study of Rolland-Cachera et al., [110]. They demonstrated that fat intake at 2 years of age was negatively associated with body fat—particularly abdominal fat—at the age of 20 years [110]. This is in line with recommendations against restricting fat intake and those supporting a gradual reduction in fat intake over the course of the first 3–4 years of life [1,18].

In our review, we identified excess fat intake in no more than 7 of the 50 studies from high income countries and overall, our findings, which are based on a more globally representative sample of countries, do not suggest a need for children from 1–7 y to reduce their intake of total fat. In line with this, data from the Generation R cohort also show no associations between a higher intake of total fat or SFA, MUFA, or PUFA with growth, adiposity, or cardiometabolic health when fat was consumed at the expense of carbohydrates [111]. Further studies investigating the intake of the other macronutrients by these age groups are required to provide a more holistic overview of their potential influence on body weight regulation. This is particularly important for carbohydrates, where, for example, sugar-sweetened beverages are consumed by up to half of children in this age group in certain countries [112] and are known to contribute significantly to weight gain [113] and incidence of overweight [114].

### 4.5. Total SFA

In nearly all recovered studies (92–98%), SFA intake is higher than the 8%E recommendation [17]. The few exceptions were studies in the Gambia [43] (2–3 years, local study in rural population), Bangladesh [60] (2–5 years, local study in rural population) and South Africa [44] (3–7 years, local study in informal settlement of urban area), three studies also showing a very low total fat intake (12.9 to 23.8%E). This suggests that the 8%E limit for total SFA intake may not be compatible with the achievement of total fat recommendation of at least 35%E in younger children or at least 25%E in older children. In our review, the observed total SFA intake is higher than the 10%E recommendation [21] in 69–73% of studies, notably in all European countries for which we found data, for all ages considered. These findings are in line with earlier reviews, which show mean intakes of SFA higher than 10%E for <10 year old children in Europe using National Survey data [14], in 2–10 year old children worldwide [15] and in young and school-aged children of Eastern Mediterranean countries [16]. The range of SFA intake varied from 3.4–18%E in our review, a narrower range than what was found in adults, where global saturated fat consumption ranged from 2.3–27.5%E [115].

In children, some benefits of reducing intake of SFA have been demonstrated in the context of cardiovascular health. A systematic review and meta-analysis concluded that reducing SFA intake in children aged 2–19 years significantly decreased total and LDL-cholesterol, as well as diastolic blood pressure, with no evidence of adverse effects on growth or development [116]. However, the wide age range of participants of the studies included in the review of Te Morenga et al., as well as the fact that five of the eight studies included subjects with hyperlipidemia, make it difficult to translate these findings to healthy toddlers and young children. Although cardiometabolic disease typically presents in later life, atherosclerotic plaques have been shown to appear in childhood [117] and are positively associated with dyslipidemia [118]. Elevated LDL-cholesterol in childhood has been associated with increased CVD risk factors in adulthood [118], however, there is no evidence of an effect of reducing SFA in the younger age group on later CVD risk.

The association between total SFA intake and health is increasingly questioned and a beneficial effect of dairy SFA on the most prevalent chronic diseases in adults, such as diabetes, has been suggested [111,119]. Recently, the SACN updated their recommendations for SFA intake in the UK and concluded that the maximum limit of 10%E for SFA intake does not apply before 2 years and applies, in full, from five years [24]. Thus, younger children may safely consume a higher proportion of their dietary fat as SFA, as a transition from full breast milk feeding to an adult diet. Then, from about 5 years of age, it may be important to keep SFA intake as low as possible to promote lifelong cardiometabolic health.

### 4.6. Essential Fatty Acids: Linoleic Acid and Alpha-Linolenic Acid

LA intake is above the lower FAO/WHO AI for all age ranges in all studies, except in 4 out of 12 studies done in 1–2 year old children (one national survey in France [78] and three local studies in Canada [49], China [61] and Australia [96], for which intake was too low. The EFSA AI for LA, based on LA intake in healthy adults in Europe, is higher than that of FAO/WHO, and LA intake was lower than the EFSA recommendations in about half of the studies for all ages considered, without specificity for geography. Sunflower seed oil contains 80–90% LA and is the most consumed vegetable oil in Turkey [120], which may explain the exceptionally high LA intake in children in this country. However, we did not have access to the original data of this survey to be able to confirm this speculation. At present, the health impact of high LA in early life is unclear. While available preclinical evidence suggests a risk for obesity and worse metabolic outcomes in adulthood in response to high LA (or high LA:ALA ratio) in early life, supportive clinical data are lacking (reviewed in [121]). Future, well-designed studies are warranted to untangle the complex relationship between these FA in early life and their role in long-term health.

ALA intake is lower than the EFSA and FAO/WHO AI in more than half of the studies for all ages considered, without specificity for geography. To the best of our knowledge, no previous reviews have compared LA and ALA intake among children to the new FAO/WHO recommendation, however, Sioen et al., [13] compared intakes in European children of these two essential FA with the EFSA AI and found that in most of studies, LA and ALA intake were lower than the AI (75% of studies in 1–3 years old for both FA; 66% [LA] and 100% [ALA] of studies in 4–9 years old). These findings were based on only 7 studies available at that time in Europe. Our findings for Europe, based on more than 15 studies, most of them published since 2017, are less pessimistic, with LA and ALA intakes being in line with European AI in about 50% of studies.

In the review of Sioen, as in our own review, LA and ALA intake are probably overestimated, because several of the recovered studies assessed n-6 PUFA (and not just LA [12 studies]) or n-3 PUFA (and not just ALA [8 studies]). It is estimated that LA represents ~97–99% of the dietary n-6 PUFA, while ALA generally represents ~80–90% of the dietary n-3 PUFA [13,54,73]. Thus, the overestimation is higher for ALA than for LA, reinforcing the finding that ALA inadequate intake is more prevalent than that of LA. While LA and ALA are the two EFA needed for normal growth and development of children [122], according to EFSA, intakes of LA above 1% do not show any additional benefits.

### 4.7. Arachidonic Acid

Currently, there is no recommendation for ARA intake in infants older than 6 months, because despite its presence in human milk, and its rapid accretion in body cells in young children, no clinical effect could be observed when infants’ diets were supplemented with ARA [26,27]. ARA intake was reported in several studies included in this review [40,43,49,51,52,56,60,61,63,73,77,78,79,89] and ranged from 10 to 260 mg per day. By comparison, fully breastfed infants aged 0–6 months consume 140 mg/day [19].

### 4.8. EPA and DHA

Intake of EPA and DHA was lower than the recommendation in nearly all studies and for all ages considered, in line with earlier review findings in children [12,13,15] and in adults [115]. N-3 LC-PUFA are highly important for brain maturation and development [3], therefore, this finding is a concern. The exceptions to this were for younger children (1–3 years) in three small studies from coastal areas (Singapore, [69], Vancouver, Canada [49,52]) and for children 3–5 years in five studies (two local (Vancouver [49], Tokyo [63], one sub-national (urban areas in Spain) [89] and two national studies (France [79], Sweden [91]), where EPA + DHA intakes were within the FAO/WHO recommendations, but lower than EFSA recommendations.

#### 4.8.1. Distribution of Intakes within the Population

Several studies reported median intakes in addition to the mean intakes. We observed that for intakes of DHA and EPA, means were much higher than medians and differ from medians more than the other PUFA. EPA and DHA intake in populations are generally not normally distributed and skewed to the right due to higher intake of few individuals [36,106]. This is mainly because not all products rich in n-3 fatty acids were consumed by all participants, while the other PUFA are more evenly distributed in foods. Future studies should aim to include median and mean DHA values to allow for a better comparison across studies and geographical regions.

#### 4.8.2. Effect of Methodology (Dietary Assessment, Food Composition Database, Inclusion of Fish Oil Supplement in the Assessment) on the Results for EPA and DHA

Among the 65 studies included in this review, only six (Table S3) reported n-3 PUFA or fish oil supplement use among their participants. The low proportion of children consuming these supplements (from 0% in Belgium [40] to 3% in Australia [36]) indicates that systematic inclusion of PUFA supplements in the dietary assessment would not greatly alter the global conclusion of our review.

Several studies from North America and Europe, which had as one objective to assess n-3 PUFA intake, used a methodology more adapted to this parameter [35,39,41,50,51,56]: they assessed the diet with FFQ combined with recalls or records; they added to the dietary assessment a FFQ specific for fish intake; they used or developed food composition databases accurate for fatty acids and three of them included fish oil supplements in the assessment [35,39,41]. Surprisingly, results of EPA and DHA intakes obtained by these “best in class” studies were not higher than intake values found in the other studies. Thus, the low values found for EPA and DHA are not likely due to inappropriate methodologies, but rather because of lower consumption of foods containing n-3 LC-PUFA, i.e., mostly fish and shellfish.

#### 4.8.3. N-3 PUFA Supplementation

The fact that dietary intake of EPA + DHA is below recommendations in most studies, begs the question of whether supplementation or food fortification of these fatty acids would be beneficial for these age groups, using, for example, fortified milks. Indeed, Madrigal et al., found a better adherence to n-3 FA recommendations in a group of children consuming adapted milks (including follow-on formula, toddler’s milk, growing-up milk, and fortified and enriched milks) [89].

Many observational and intervention studies explored the effects of n-3 LC PUFA among this age group and the vast majority of DHA supplementation trials to-date in children have focused on brain function outcomes, likely due to the known influence of DHA on brain development in early life [123]. A recent systematic review of n-3 PUFA supplementation in relation to cognition in children and adolescents (4–25 y) concluded that it seemed that an increase in the circulating levels of n-3 LCPUFA, reaching >6% EPA + DHA in blood erythrocytes, was required for a positive effect on cognition and that in healthy children, daily supplementation with at least 450 mg DHA + EPA was necessary for this improvement [124].

Another area of research is the influence of n-3 PUFA supplementation on body composition and obesity risk in children, however, few studies exist beyond the pregnancy/lactation period. Even during this period, at present, evidence does not support a benefit of n-3 PUFA supplementation for later obesity risk reduction in infants and young children [125,126]. In older children (7–12 y), dietary intakes of n-3 PUFA are positively related to lean mass accretion [127], but further research is required to determine the impact of n-3 PUFA supplementation at this stage in life on body composition and cardiometabolic health, both in the short- and long-term.

### 4.9. Studies with All Parameters Outside of the Recommended Range

In a few studies, the intakes of all parameters (total fat, SFA, LA, ALA, DHA) were outside the recommended ranges, being lower (or higher for SFA) than the recommendations. It was observed e.g., in a rural population in China [61], in national surveys in France [78], Canada [48] or in Ireland [83]. This highlights that inadequate intake of these fatty acids can occur locally, such as in a poor, rural population in China, but also on a national level, such as those studies conducted in developed countries.

### 4.10. Strengths and Limitations

Unlike previous reviews which tended to focus on one geographic region, a strength of the current work lies in the fact that it provides a global overview of fat intake among children. A second merit is that this review provides information on individual PUFA (LA, ALA, EPA, DHA) and SFA intakes, and not just total fat or total PUFA intakes. Additionally, this review employed a detailed age stratification (1–2, 2–3, 3–5 and 5–7 years), allowing to highlight the low intake of total fat in the younger children (1–3 years), the low LA intake in the younger group (1–2 years) and more specifically, the low intake of EPA + DHA in the younger (1–2 years) and the older (5–7 years) sub-groups. This stratification may help for a more targeted approach to assessing intake and developing interventions for particular age groups. A limitation of the current review is that it did not employ systematic search methods. Also, there is substantial heterogeneity in the methodologies employed by the included studies to assess dietary intake, food databases were not all the same quality and the age grouping differed between countries. Furthermore, about 30% of the included studies were local studies, and it is possible that a high proportion of the volunteer participants were concerned about their diet, which may not be entirely representative of the total population. The interpretations made in this paper are dependent on the available guidelines, which themselves have certain limitations. For example, the FAO/WHO guidelines for total fat, total SFA, LA, ALA, MUFA and total PUFA intake in children cover a wide age range (2–18 years), while the EFSA guideline for SFA is “as low as possible”. These vague recommendations, when considered in isolation, make it challenging to compare observed vs. recommended intakes. However, an advantage of the current study is that it takes into consideration both FAO/WHO and EFSA guidelines, as well as recent literature in the field, to generate cut-offs relevant for each fatty acid within each age group, thus providing more precise values against which intakes were compared.

## 5. Conclusions

Overall, these findings highlight suboptimal total fat intake among younger children (1–3 y), while fat intakes were either too low, adequate or higher than recommended for >3-year-olds (in ~12%, 70% and 19% of the studies, respectively). Intake of SFA was generally above recommended maximum values for all ages assessed, particularly in European countries. While intake of LA may be a concern for younger children (1–2 years), it is within the recommended FAO/WHO range after that age. The intake of ALA is below the recommendations in greater than half of all studies for all age ranges considered. The intake of n-3 LC PUFA among children was suboptimal across all age groups in most countries worldwide. Future, well-designed studies are warranted, particularly from Africa, South America, China and India, where there is a scarcity of data. This will help to clarify these findings and to identify the need for specific public health measures to increase intake of important fatty acids in the diets of young children, who make up a significant proportion of the population in these countries and for whom these fatty acids are crucial for growth and development.

## Figures and Tables

**Table 1:** FAO/WHO dietary recommended intakes and EFSA dietary reference values for energy, total fat, SFA, LA, ALA, DHA, sum of EPA+DHA, total MUFA and total PUFA for children aged 1–2 years, 2–3 years, 3–5 years and 5–7 years.

Ref.	Age Groups	Energy (kcal)	Total Fat	SFA	LA	ALA	DHA	EPA + DHA	MUFA	PUFA
FAO/WHO 2010 [17]	1–2 y	900	Gradual reduction, depending on physical activity, to 35%E (AMDR)	No value for this age group	1–2 y: 3–4.5%E (AI)	6–24 months: 0.4–0.6%E (AI)	6–24 months: 10–12 mg/kg (AI)	6–24 months No DRV	6–24 months No DRV	6–24 months No AI; <15%E (U-AMDR)
FAO/WHO 2010	2–3 y	1100	2–18 y: 25–35%E (AMDR)	2–18 y: 8%E (U-AMDR)	>2 y: 2–3%E (AI) 2% (EAR) 2.5–9%E (AMDR)	>2 y: >0.5%E (L-AMDR) 0.5–2%E (AMDR)	No DRV	2–4 y: 100–150 mg (AI)	2–18 y: Total fat—SFA—PUFA—TFA (AMDR)	2–18 y: No AI 11%E (U-AMDR)
FAO/WHO 2010	3–5 y	1250	2–18 y: 25–35%E (AMDR)	2–18 y: 8%E (U-AMDR)	>2 y: 2–3%E (AI) 2% (EAR) 2.5–9%E (AMDR)	>2 y: >0.5%E (L-AMDR) 0.5–2%E (AMDR)	No DRV	2–4 y: 100–150 mg (AI) 4–6 y: 150–200 mg (AI)	2–18 y: Total fat—SFA—PUFA—TFA (AMDR)	2–18 y: No AI 11%E (U-AMDR)
FAO/WHO 2010	5–7 y	1450	2–18 y: 25–35%E (AMDR)	2–18 y: 8%E (U-AMDR)	>2 y: 2–3%E (AI) 2% (EAR) 2.5–9%E (AMDR)	>2 y: >0.5%E (L-AMDR) 0.5–2%E (AMDR)	No DRV	4–6 y: 150–200 mg (AI) 6–10 y: 200–250 mg (AI)	2–18 y: Total fat—SFA—PUFA—TFA (AMDR)	2–18 y: No AI 11%E (U-AMDR)
EFSA 2010 [18] 2013 [19]	1–2 y	865.75 ^1^	1–3.9 y: 35–40%E (RI)	“As low as possible”	4%E (AI)	0.5%E (AI)	1–2 y: 100 mg (AI)	No DRV	No DRV	No DRV
EFSA 2010 2013	2–3 y	1061 ^2^	1–3.9 y: 35–40%E (RI)	“As low as possible”	4%E (AI)	0.5%E (AI)	No DRV	=and >2 y: 250 mg (AI)	No DRV	No DRV
EFSA 2010 2013	3–5 y	1329.5 ^3^	1–3.9 y: 35–40%E (RI) =and >4 y: 20–35%E (RI)	“As low as possible”	4%E (AI)	0.5%E (AI)	No DRV	=and >2 y: 250 mg (AI)	No DRV	No DRV
EFSA 2010 2013	5–7 y	1560 ^4^	=and >4 y: 20–35%E (RI)	“As low as possible”	4%E (AI)	0.5%E (AI)	No DRV	=and >2 y: 250 mg (AI)	No DRV	No DRV

Abbreviations: AI: adequate intake; ALA: α-linolenic acid; AMDR: acceptable macronutrient distribution range; DHA: docosahexaenoic acid; DRV: dietary reference values; EAR: estimated average requirements; EFSA: European Food Safety Authority; EPA: eicosapentaenoic acid; FAO: Food and Agriculture Organization; L-AMDR: lower acceptable macronutrient distribution range; LA: linoleic acid; MUFA: monounsaturated fatty acids; PUFA: polyunsaturated fatty acids; RI: recommended intake; SFA: saturated fatty acids; U-AMDR: upper acceptable macronutrient distribution range; TFA: *trans* fatty acids; WHO: World Health Organization; y: years. ^1^ total average daily energy requirement for boys and girls of 866 kcal for 1–2 year old children (using a low physical activity level of 1.4) (mean of 744.5 kcal/day for 1 year old boys and girls and 987 kcal/day for 2 year old boys and girls) [19,20]. ^2^ total average daily energy requirement for boys and girls of 1061 kcal for 2–3 year old children (using a low physical activity level of 1.4)(mean of 987 kcal/day for 2 year old boys and girls and 1135 kcal/day for 3 year old boys and girls) [19,20]. ^3^ total average daily energy requirements for boys and girls of 1329.5 kcal for 3–5 years (using a low physical activity level of 1.4 for 3 years and a moderate level of 1.6 for 4 and 5 years) [20]. ^4^ total average daily energy requirement for boys and girls aged 5 years, 6 years and 7 years (using a moderate physical activity level of 1.6) [20].

**Table 3:** Recommended cut-off values and summary of the evaluation against the recommendation (values are: number of studies not in line with recommendations/total number of studies and % studies not in line with recommendations).

Fat or FA	Recommendation	1–2 y	2–3 y	3–5 y	5–7 y
Total fat	1–2 y: Min. 35% (Min. FAO/WHO and Min. EFSA) 2–3 y: Min. 35%E (Max. FAO/WHO and Min. EFSA) 3–5 y: 25–35%E (FAO/WHO and mid-range of EFSA) 5–7 y: 25–35% (FAO/WHO and EFSA for upper end of the range)	23/26 (88%) too low	25/28 (89%) too low	5.25 */33 (16%) too low 5.5/33 (17%) too high	3/34 (9%) too low 6.5/34 (19%) too high
SFA	FAO/WHO 2010: Max. 8%E	No recommendation	24/26 (92%) too high	30.75/32 (96%) too high	32.5/33 (98%) too high
SFA	FAO/WHO 2019: Max 10%E	No recommendation	19/26 (73%) too high	22/32 (69%) too high	24/33 (73%) too high
LA	FAO/WHO: 1–2 y: Min 3%E >2 y: Min 2%	4/17 (24%) too low	0/18 0%	0/22 0%	0/18 0%
LA	EFSA: 4%E	10/17 (59%) too low	8/18 (44%) too low	9/22 (41%) too low	9/18 (50%) too low
ALA	0.5%E	8/19 (42%) too low	10/20 (50%) too low	10/24 (42%) too low	8/19 (42)% too low
DHA or EPA + DHA	FAO/WHO: DHA 1–2 y: Min 100 mg EPA+DHA 2–3 y: Min 100 mg 3–5 y: Min 125 mg 5–7 y: Min. 175 mg	12/13 (92%) too low	7/10 (70%) too low	9/14 (64%) too low	11/11 (100%) too low
DHA or EPA + DHA	EFSA DHA 1–2 y: 100 mg EPA+DHA >2 y: 250 mg	12/13 (92%) too low	10/10 (100%) too low	14/14 (100%) too low	11/11 (100%) too low

Abbreviations: y: year; ALA: alpha-linolenic acid; DHA: docosahexaenoic acid; EFSA: European Food Safety Authority; EPA: eicosapentaenoic acid; FAO: Food and Agriculture Organisation; LA: linoleic acid; SFA: saturated fatty acids; WHO: World Health Organisation. * A few studies were counted as half or quarter if some of the subjects within the study met the recommendations, but some subjects did not e.g., boys (1/2 study) met recommendations, girls (1/2 study) did not.

## Supplementary Materials

The following are available online at https://www.mdpi.com/article/10.3390/nu13103547/s1, Figure S1: Mean/median total fat intake (percentage total daily energy intake [%E]) for children aged 1–2 years; 2–3 years; 3–5 years; 5–7 years. Figure S2: Mean/median total SFA intake (percentage total daily energy intake [%E]) for children aged 2–3 years; 3–5 years; 5–7 years. Figure S3: Mean/median linoleic acid (LA) intake (percentage total daily energy intake [%E]) for children aged 1–2 years; 2–3 years; 3–5 years; 5–7 years. Figure S4: Mean/median alpha-linolenic acid (ALA) intake (percentage total daily energy intake [%E]) for children aged 1–2 years; 2–3 years; 3–5 years; 5–7 years. Figure S5: Mean/median docosahexaenoic acid (DHA) and sum of DHA + eicosapentaenoic acid (EPA) intake (mg/day) for children aged 1–2 years; 2–3 years; 3–5 years; 5–7 years. Table S1: Objectives and representativeness of the included studies. Table S2: Dietary assessment methods used in the included studies. Table S3: Record of supplements, fortified foods and breast milk consumption in the included studies. Table S4: Daily intake of total fat, linoleic acid, alpha-linolenic acid (%E) and docosahexaenoic acid (mg) in children aged 1 to 2 years (mean or median for boys and girls unless otherwise specified). Table S5: Daily intake of total fat, saturated fats, linoleic acid, alpha-linolenic acid (%E) and sum of docosahexaenoic + eicosapentaenoic acids (mg) in children aged 2 to 3 years (mean or median for boys and girls unless otherwise specified). Table S6: Daily intake of total fat, saturated fats, linoleic acid, alpha-linolenic acid (%E) and docosahexaenoic + eicosapentaenoic acids (mg) in children aged 3 to 5 years (mean or median for boys and girls unless otherwise specified). Table S7: Daily intake of total fat, saturated fats, linoleic acid, alpha-linolenic acid (%E) and docosahexaenoic + eicosapentaenoic acids (mg) in children aged 5 to 7 years (mean or median for boys and girls unless otherwise specified).
