# Supplementary material for: Total Fat and Fatty Acid Intake among 1–7-Year-Old Children from 33 Countries: Comparison with International Recommendations
Source: Nutrients. 2021 Oct 9;13(10):3547. doi: 10.3390/nu13103547 (PMC8537778; doi:10.3390/nu13103547)
Supplement: Supplementary file 1 [file nutrients-13-03547-s001.zip › Supplementary_Figures_S1_to_S5_Monnard_Fleith.pdf]

Supplementary Figure Legends:
Values used to build the figures can be found in Tables S4 to S7; when the study provided more than one intake value for a given fatty acid and a given age (e.g. a value for boys and a value for girls) the worst case value (i.e. the value which was the furthest from the recommended value) was used for the figure.

Figure S1: Mean/ median total fat intake (percentage total daily energy intake [%E]) for children aged (A) 1-2 years; (B) 2-3 years; (C) 3-5 years; (D) 5-7 years.

Figure S2: Mean/ median total SFA intake (percentage total daily energy intake [%E]) for children aged (A) 2-3 years; (B) 3-5 years; (C) 5-7 years.

Figure S3: Mean / median linoleic acid (LA) intake (percentage total daily energy intake [%E])]) for children aged (A) 1-2 years; (B) 2-3 years; (C) 3-5 years; (D) 5-7 years.

Figure S4: Mean/ median alpha-linoleic acid (ALA) intake (percentage total daily energy intake [%E])]) for children aged (A) 1-2 years; (B) 2-3 years; (C) 3-5 years; (D) 5-7 years.

Figure S5: Mean/ median docosahexaenoic acid (DHA) and sum of DHA + eicosapentaenoic acid (EPA) intake (mg/ day)]) for children aged (A) 1-2 years; (B) 2-3 years; (C) 3-5 years; (D) 5-7 years.

Figure S1

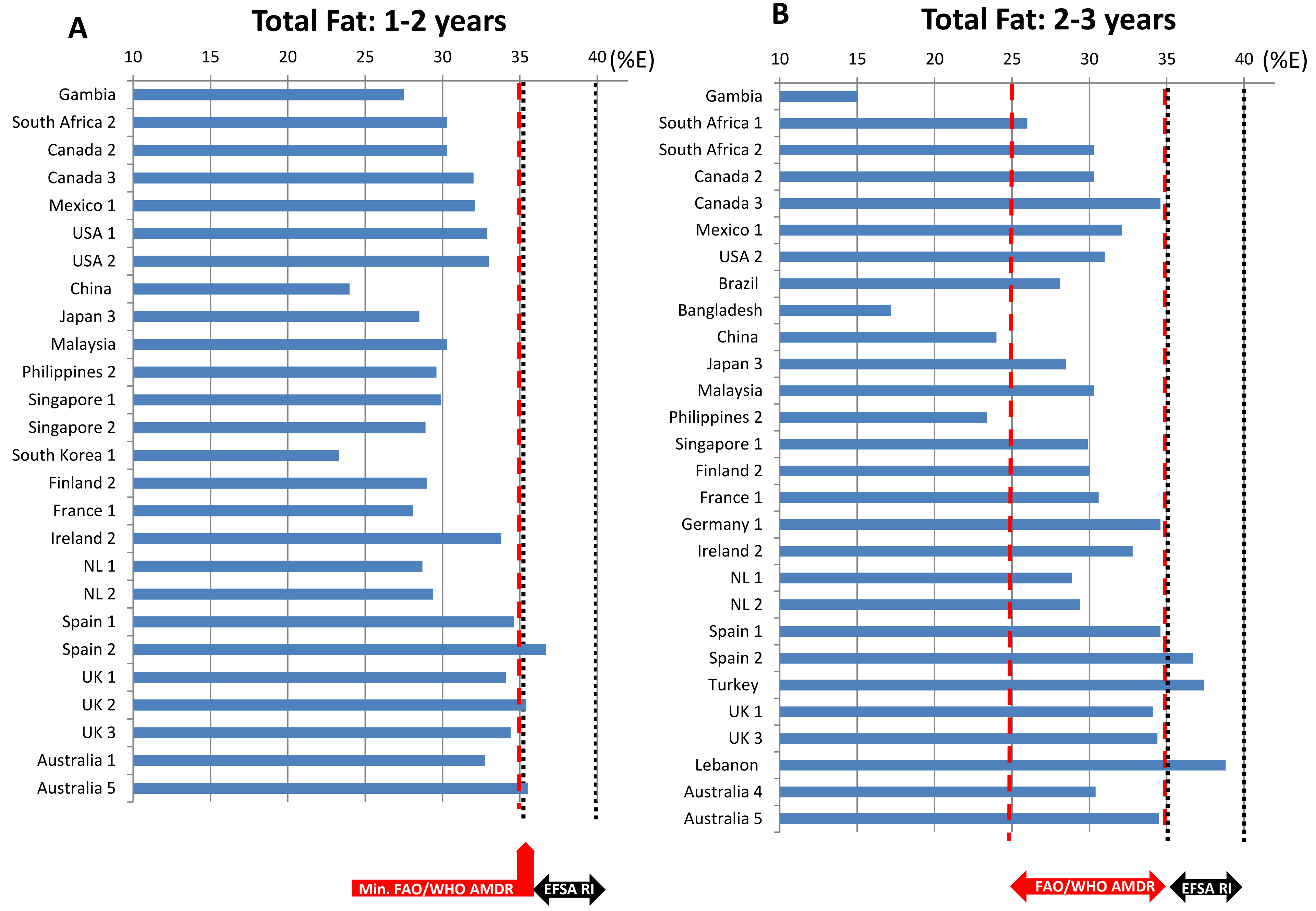

A
Total Fat: 1-2 years
10
15
20
25
30
35
40
(%E)
Gambia
South Africa 2
Canada 2
Canada 3
Mexico 1
USA 1
USA 2
China
Japan 3
Malaysia
Philippines 2
Singapore 1
Singapore 2
South Korea 1
Finland 2
France 1
Ireland 2
NL 1
NL 2
Spain 1
Spain 2
UK 1
UK 2
UK 3
Australia 1
Australia 5
Min. FAO/WHO AMDR
EFSA RI
B
Total Fat: 2-3 years
10
15
20
25
30
35
40
(%E)
Gambia
South Africa 1
South Africa 2
Canada 2
Canada 3
Mexico 1
USA 2
Brazil
Bangladesh
China
Japan 3
Malaysia
Philippines 2
Singapore 1
Finland 2
France 1
Germany 1
Ireland 2
NL 1
NL 2
Spain 1
Spain 2
Turkey
UK 1
UK 3
Lebanon
Australia 4
Australia 5
FAO/WHO AMDR
EFSA RI

Figure S1

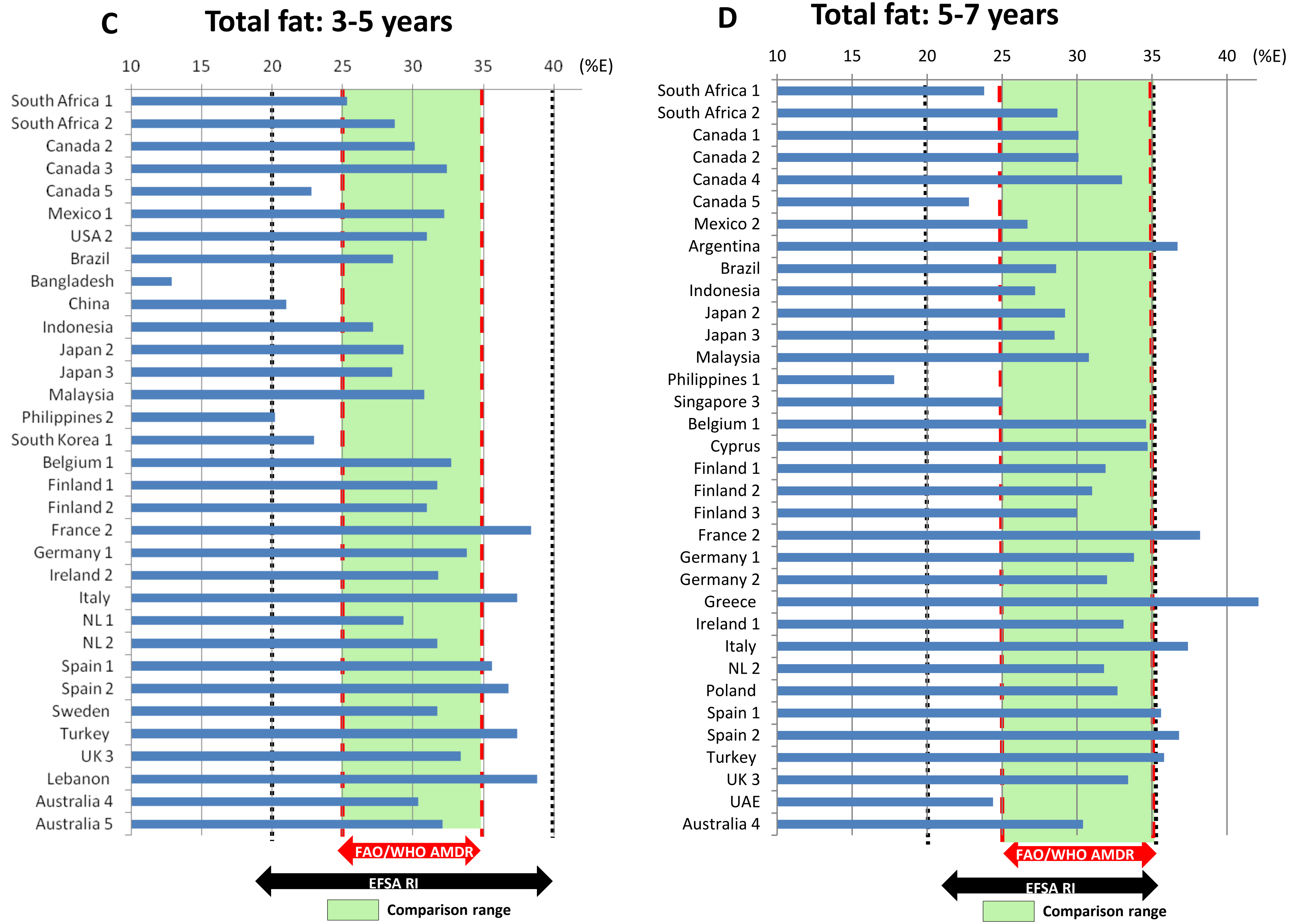
C
Total fat: 3-5 years
10
15
20
25
30
35
40
(%E)
South Africa 1
South Africa 2
Canada 2
Canada 3
Canada 5
Mexico 1
USA 2
Brazil
Bangladesh
China
Indonesia
Japan 2
Japan 3
Malaysia
Philippines 2
South Korea 1
Belgium 1
Finland 1
Finland 2
France 2
Germany 1
Ireland 2
Italy
NL 1
NL 2
Spain 1
Spain 2
Sweden
Turkey
UK 3
Lebanon
Australia 4
Australia 5
FAO/WHO AMDR
EFSA RI
Comparison range
D
Total fat: 5-7 years
10
15
20
25
30
35
40
(%E)
South Africa 1
South Africa 2
Canada 1
Canada 2
Canada 4
Canada 5
Mexico 2
Argentina
Brazil
Indonesia
Japan 2
Japan 3
Malaysia
Philippines 1
Singapore 3
Belgium 1
Cyprus
Finland 1
Finland 2
Finland 3
France 2
Germany 1
Germany 2
Greece
Ireland 1
Italy
NL 2
Poland
Spain 1
Spain 2
Turkey
UK 3
UAE
Australia 4
FAO/WHO AMDR
EFSA RI
Comparison range

Figure S2

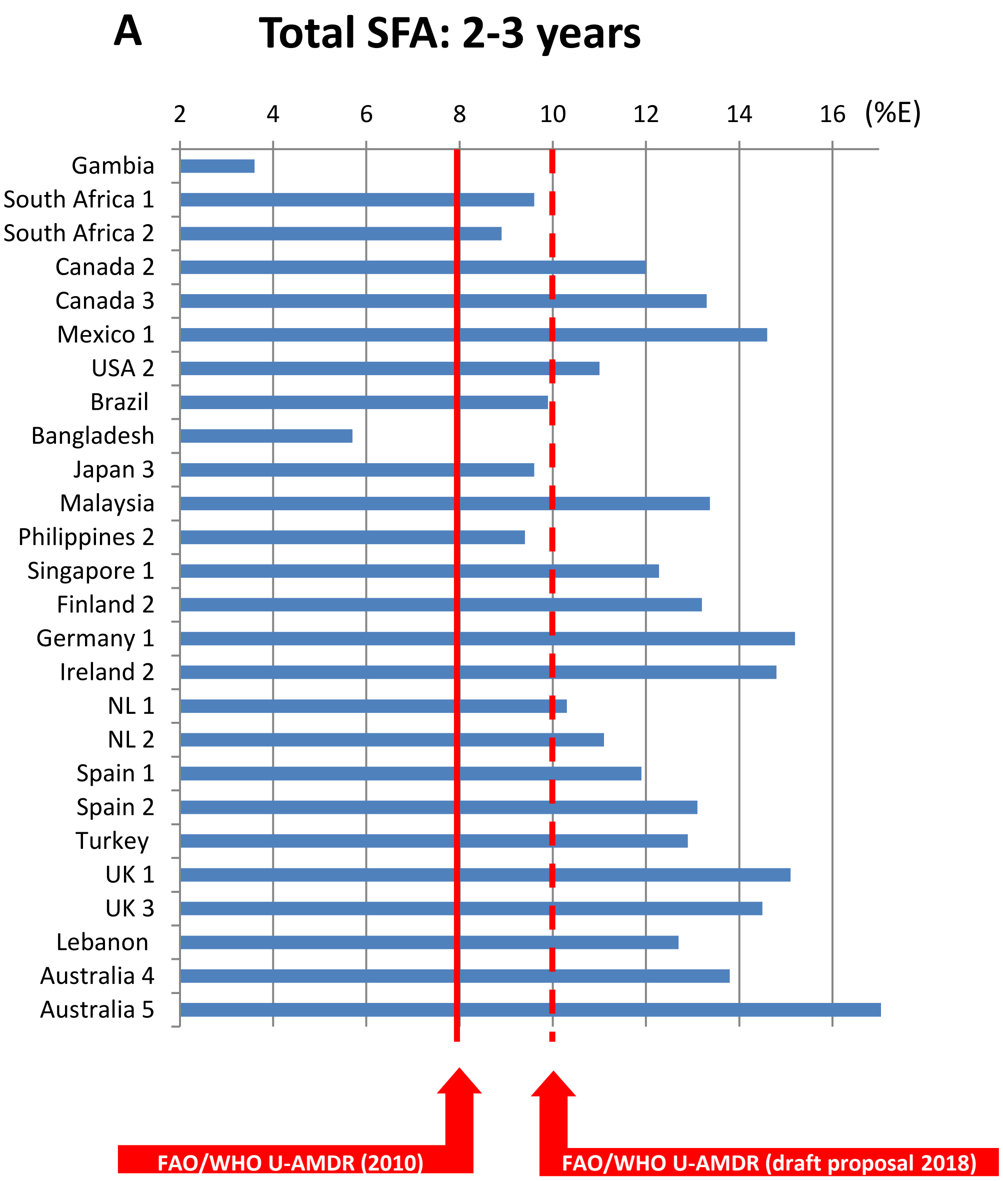

A
Total SFA: 2-3 years
2
4
6
8
10
12
14
16
(%E)
Gambia
South Africa 1
South Africa 2
Canada 2
Canada 3
Mexico 1
USA 2
Brazil
Bangladesh
Japan 3
Malaysia
Philippines 2
Singapore 1
Finland 2
Germany 1
Ireland 2
NL 1
NL 2
Spain 1
Spain 2
Turkey
UK 1
UK 3
Lebanon
Australia 4
Australia 5
FAO/WHO U-AMDR (2010)
FAO/WHO U-AMDR (draft proposal 2018)

Figure S2

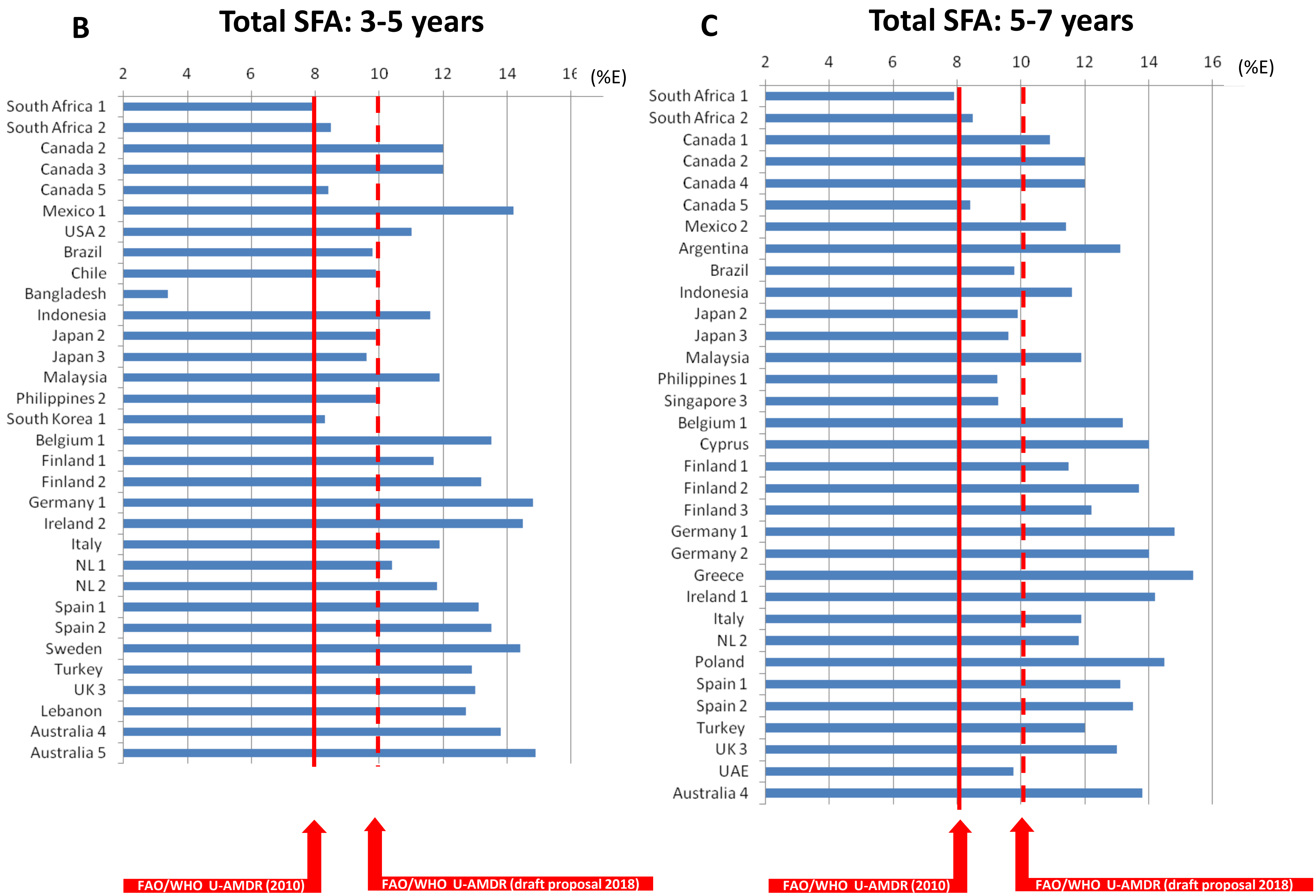
B
Total SFA: 3-5 years
2 4 6 8 10 12 14 16 (%E)
South Africa 1
South Africa 2
Canada 2
Canada 3
Canada 5
Mexico 1
USA 2
Brazil
Chile
Bangladesh
Indonesia
Japan 2
Japan 3
Malaysia
Philippines 2
South Korea 1
Belgium 1
Finland 1
Finland 2
Germany 1
Ireland 2
Italy
NL 1
NL 2
Spain 1
Spain 2
Sweden
Turkey
UK 3
Lebanon
Australia 4
Australia 5
FAO/WHO U-AMDR (2010)
FAO/WHO U-AMDR (draft proposal 2018)
C
Total SFA: 5-7 years
2 4 6 8 10 12 14 16 (%E)
South Africa 1
South Africa 2
Canada 1
Canada 2
Canada 4
Canada 5
Mexico 2
Argentina
Brazil
Indonesia
Japan 2
Japan 3
Malaysia
Philippines 1
Singapore 3
Belgium 1
Cyprus
Finland 1
Finland 2
Finland 3
Germany 1
Germany 2
Greece
Ireland 1
Italy
NL 2
Poland
Spain 1
Spain 2
Turkey
UK 3
UAE
Australia 4
FAO/WHO U-AMDR (2010)
FAO/WHO U-AMDR (draft proposal 2018)

Figure S3

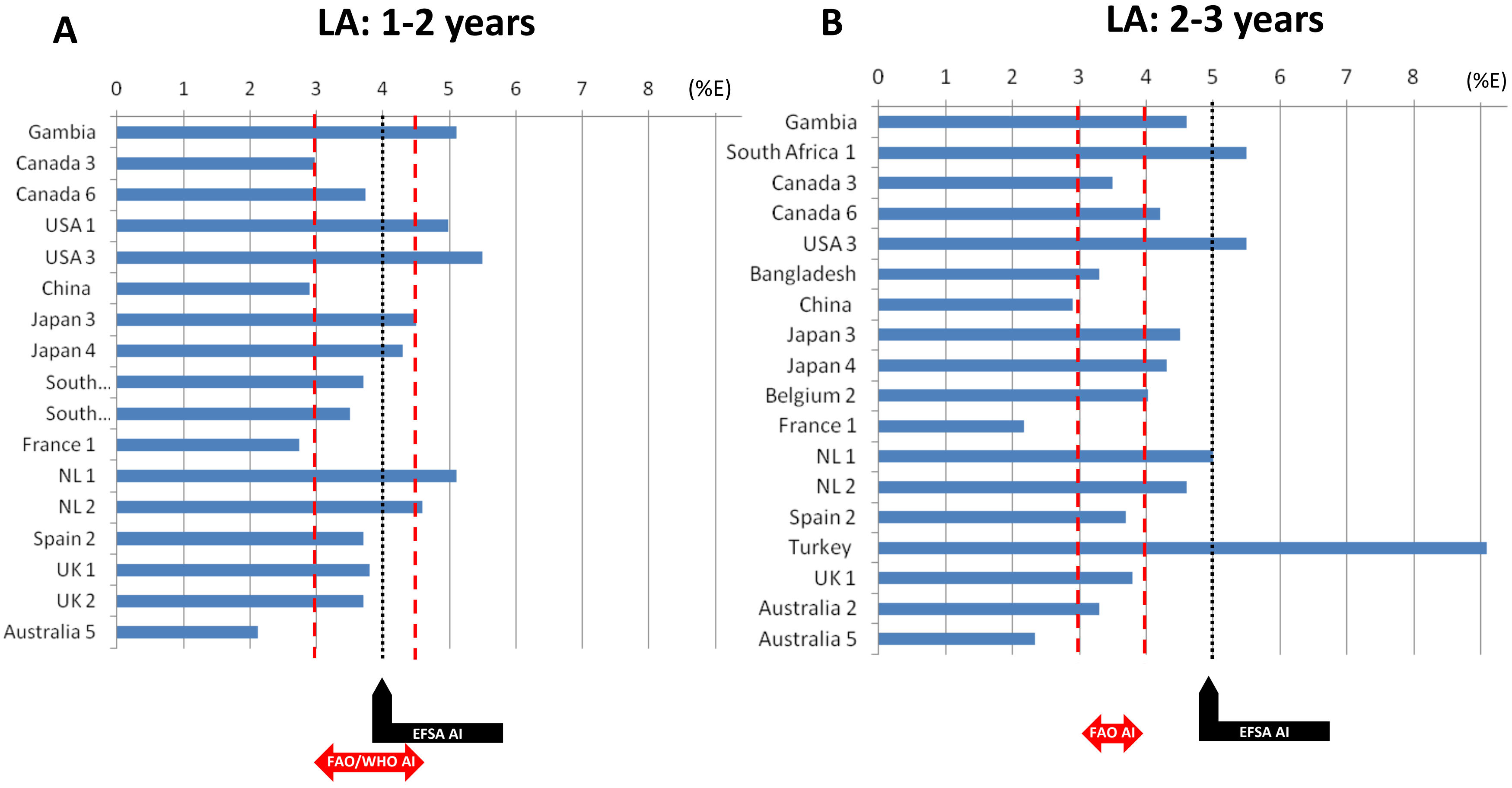
A
LA: 1-2 years
0 1 2 3 4 5 6 7 8 (%E)
Gambia
Canada 3
Canada 6
USA 1
USA 3
China
Japan 3
Japan 4
South...
South...
France 1
NL 1
NL 2
Spain 2
UK 1
UK 2
Australia 5
EFSA AI
FAO/WHO AI
B
LA: 2-3 years
0 1 2 3 4 5 6 7 8 (%E)
Gambia
South Africa 1
Canada 3
Canada 6
USA 3
Bangladesh
China
Japan 3
Japan 4
Belgium 2
France 1
NL 1
NL 2
Spain 2
Turkey
UK 1
Australia 2
Australia 5
FAO AI
EFSA AI

Figure S3

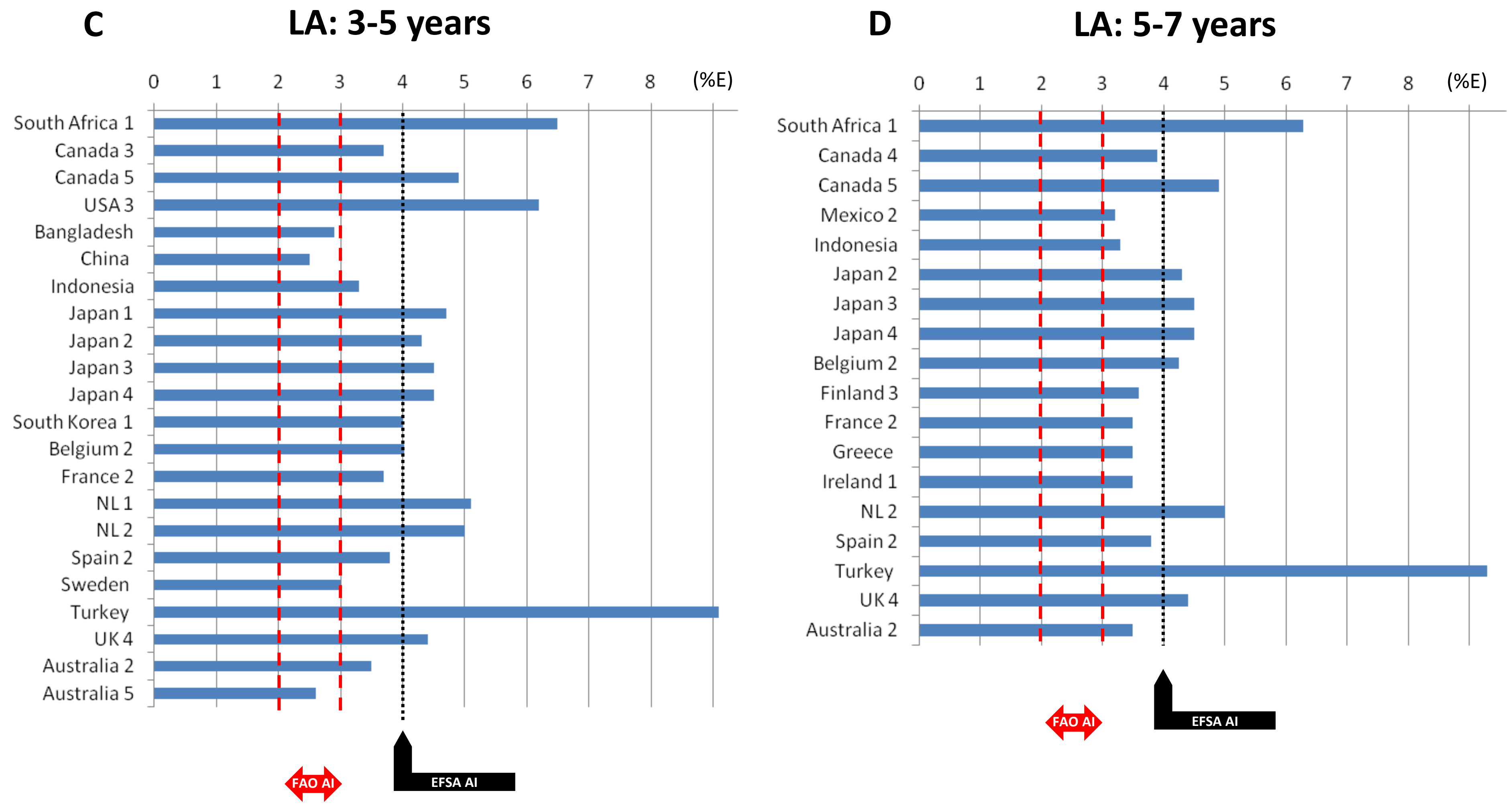
C
LA: 3-5 years
0 1 2 3 4 5 6 7 8 (%E)
South Africa 1
Canada 3
Canada 5
USA 3
Bangladesh
China
Indonesia
Japan 1
Japan 2
Japan 3
Japan 4
South Korea 1
Belgium 2
France 2
NL 1
NL 2
Spain 2
Sweden
Turkey
UK 4
Australia 2
Australia 5
FAO AI
EFSA AI
D
LA: 5-7 years
0 1 2 3 4 5 6 7 8 (%E)
South Africa 1
Canada 4
Canada 5
Mexico 2
Indonesia
Japan 2
Japan 3
Japan 4
Belgium 2
Finland 3
France 2
Greece
Ireland 1
NL 2
Spain 2
Turkey
UK 4
Australia 2
FAO AI
EFSA AI

Figure S4

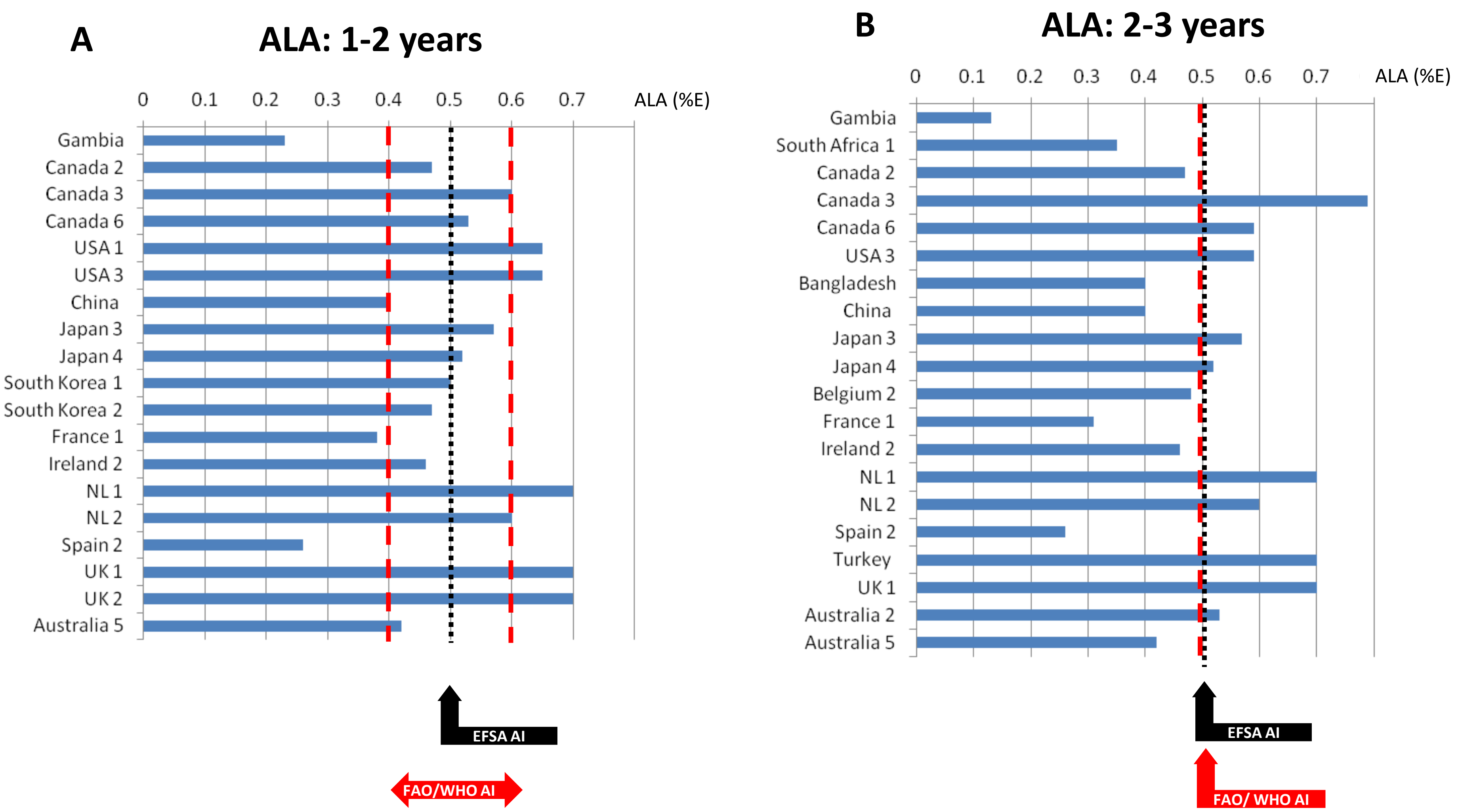
A
ALA: 1-2 years
0
0.1
0.2
0.3
0.4
0.5
0.6
0.7
ALA (%E)
Gambia
Canada 2
Canada 3
Canada 6
USA 1
USA 3
China
Japan 3
Japan 4
South Korea 1
South Korea 2
France 1
Ireland 2
NL 1
NL 2
Spain 2
UK 1
UK 2
Australia 5
EFSA AI
FAO/WHO AI
B
ALA: 2-3 years
ALA (%E)
Gambia
South Africa 1
Canada 2
Canada 3
Canada 6
USA 3
Bangladesh
China
Japan 3
Japan 4
Belgium 2
France 1
Ireland 2
NL 1
NL 2
Spain 2
Turkey
UK 1
Australia 2
Australia 5
EFSA AI
FAO/ WHO AI

Figure S4

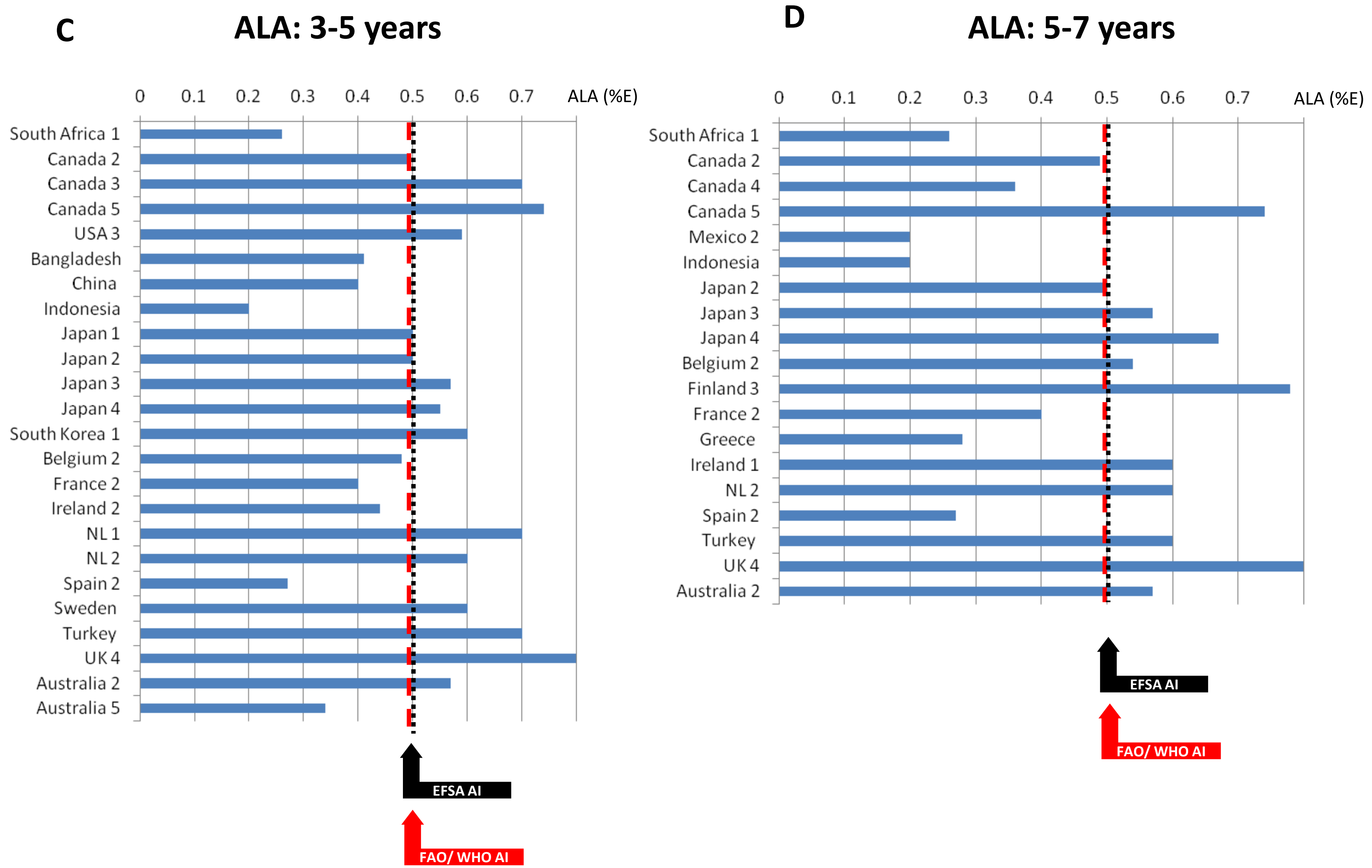
C
ALA: 3-5 years
0
0.1
0.2
0.3
0.4
0.5
0.6
0.7
ALA (%E)
South Africa 1
Canada 2
Canada 3
Canada 5
USA 3
Bangladesh
China
Indonesia
Japan 1
Japan 2
Japan 3
Japan 4
South Korea 1
Belgium 2
France 2
Ireland 2
NL 1
NL 2
Spain 2
Sweden
Turkey
UK 4
Australia 2
Australia 5
EFSA AI
FAO/ WHO AI
D
ALA: 5-7 years
0
0.1
0.2
0.3
0.4
0.5
0.6
0.7
ALA (%E)
South Africa 1
Canada 2
Canada 4
Canada 5
Mexico 2
Indonesia
Japan 2
Japan 3
Japan 4
Belgium 2
Finland 3
France 2
Greece
Ireland 1
NL 2
Spain 2
Turkey
UK 4
Australia 2
EFSA AI
FAO/ WHO AI

Figure S5

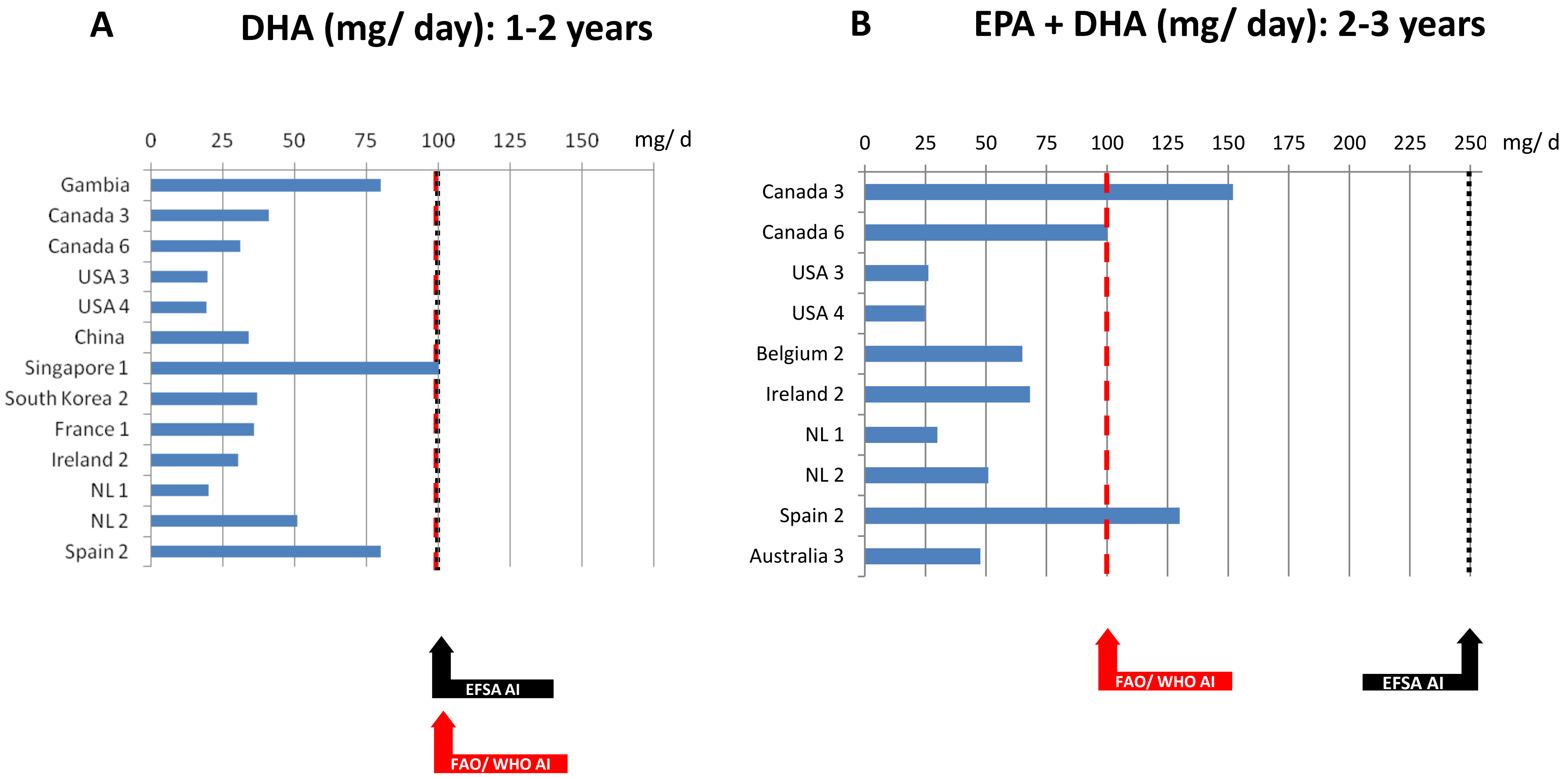
A
DHA (mg/ day): 1-2 years
0
25
50
75
100
125
150
mg/ d
Gambia
Canada 3
Canada 6
USA 3
USA 4
China
Singapore 1
South Korea 2
France 1
Ireland 2
NL 1
NL 2
Spain 2
EFSA AI
FAO/ WHO AI
B
EPA + DHA (mg/ day): 2-3 years
0
25
50
75
100
125
150
175
200
225
250
mg/ d
Canada 3
Canada 6
USA 3
USA 4
Belgium 2
Ireland 2
NL 1
NL 2
Spain 2
Australia 3
FAO/ WHO AI
EFSA AI

Figure S5

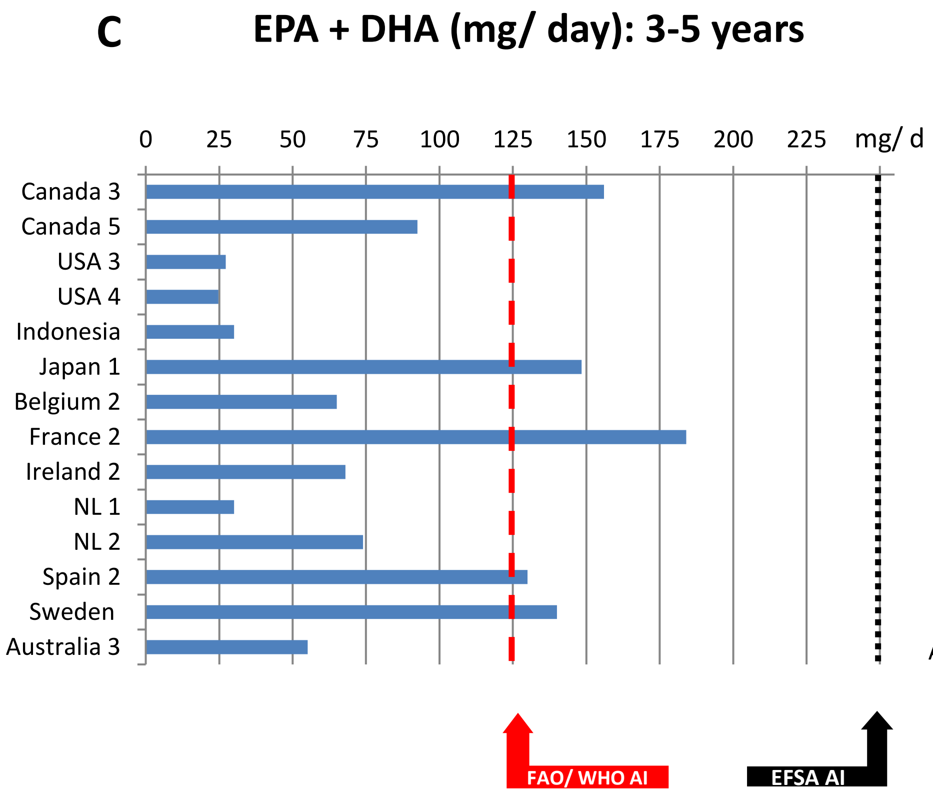
C
EPA + DHA (mg/ day): 3-5 years
0
25
50
75
100
125
150
175
200
225
mg/ d
Canada 3
Canada 5
USA 3
USA 4
Indonesia
Japan 1
Belgium 2
France 2
Ireland 2
NL 1
NL 2
Spain 2
Sweden
Australia 3
FAO/ WHO AI
EFSA AI

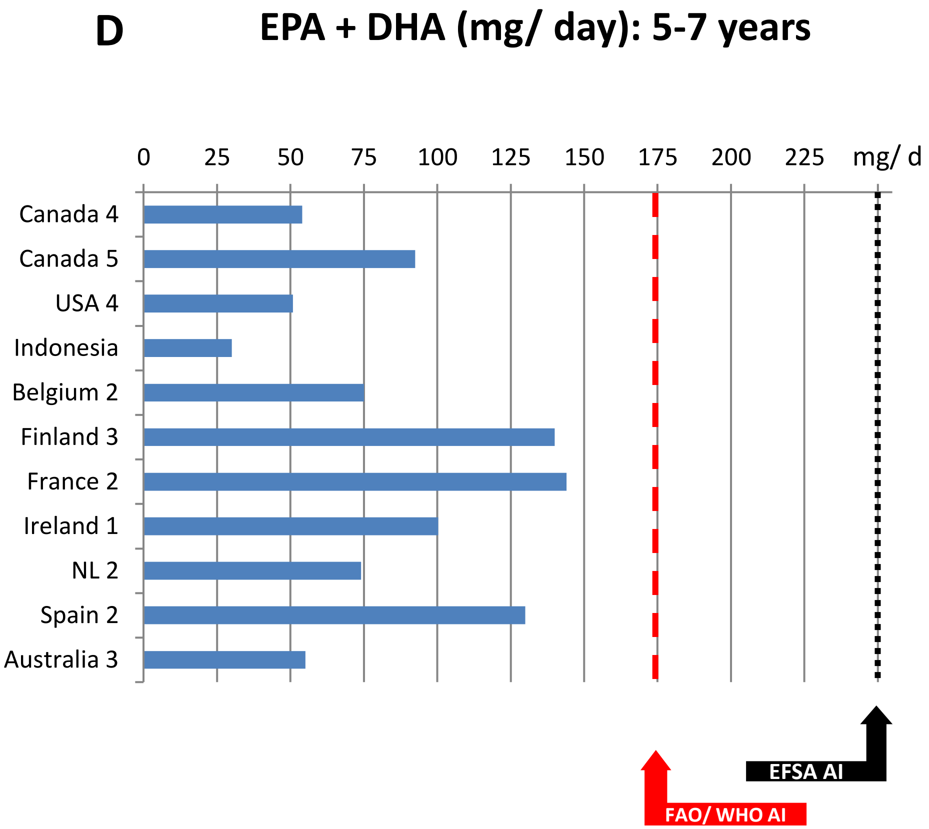
D
EPA + DHA (mg/ day): 5-7 years
0
25
50
75
100
125
150
175
200
225
mg/ d
Canada 4
Canada 5
USA 4
Indonesia
Belgium 2
Finland 3
France 2
Ireland 1
NL 2
Spain 2
Australia 3
EFSA AI
FAO/ WHO AI
