# Supplementary material for: Total Fat and Fatty Acid Intake among 1–7-Year-Old Children from 33 Countries: Comparison with International Recommendations
Source: Nutrients. 2021 Oct 9;13(10):3547. doi: 10.3390/nu13103547 (PMC8537778; doi:10.3390/nu13103547)
Supplement: Supplementary file 1 [file nutrients-13-03547-s001.zip › Supplementary_Tables_S1toS7_Monnard_Fleith.pdf]

Supplementary tables

Table S1 Objective and representativeness of the included studies*

Country	*Reference*	*Study objective*	*Local* observation study*	*Other type of local* study*	*Sub-national** observation study*	*National survey or nationwide*
Gambia	Prentice 2000	Fat and energy needs of children in developing countries	Rural			
South Africa 1	Dannhauser 2000	Nutritional status of preschool children in informal settlements		Baseline data of local intervention study (urban, stratified population sampling)		
South Africa 2	Steyn 2020	Energy and macronutrient intake in children			Urban, stratified population sampling	
Canada 1	Barr 2018	Contribution of breakfast to nutrient and food groups intake (all ages)				x
Canada 2	Health Canada 2012	Nutrient requirements satisfaction from foods (all ages)				x
Canada 3	Innis 2004	DHA and DPA n-6 PUFA intake vs. status in preschool children	Urban, day care or preschool centers			
Canada 4	Lien 2009	DHA and ARA intake in children	X			
Canada 5	Madden 2009	N-3 PUFA intake in children (chemical analysis)	X			
Canada 6	Wiedeman 2019	N-6 and n-3 PUFA intake vs. status in toddlers (longitudinal RCT)		Dietary intake data from a RCT (urban, local)		
Mexico 1	Jimenez-Aguilar 2018	Energy and nutrient intake vs. recommendations in preschool children				x
Mexico 2	Ramirez-Silva	FA intake in the population				x
USA 1	Ahluwalia 2016	Nutrients intake vs. recommendations in children				x
USA 2	Bailey 2018	Nutrient intake in children				x
USA 3	Keim 2015	PUFA and fish intake in children				x
USA 4	Thomson 2019	N-3 PUFA intake all ages				x
Argentina	Cuesta 2018	Nutrient intake and obesity in children and adolescents	Urban, stratified population sampling			

Country	*Reference*	*Study objective*	*Local* observation study*	*Other type of local* study*	*Sub-national** observation study*	*National survey or nationwide*
Brazil	Bueno 2013	Nutrient risk in children			Day care centers and schools	
Chile	Rebolledo 2019	Dietary intake by food source in children and adolescents vs. family income	Urban, preschool			
Banglade sh	Yakes 2011	PUFA intake adequacy vs. Breastfeeding in children	Rural, random sampling			
China	Barbarich 2006	FA + macronutrient intake, vs. anthropometry in rural children	Rural			
Indonesia	Neufingerl 2016	FA intake in children				x
Japan 1	Ando 2019	EPA + DHA intake vs. status in preschool children	Urban			
Japan 2	Murakami 2018b	Nutrient intake adequacy in children attending nursery				x
Japan 3	NHNS-J 2018	Nutrient intake, all population				x
Japan 4	Tsuboyama-Kasaoka 2013	Representativity of 2 y measures of nutrient intake (all ages)				x
Malaysia	Mohd Shariff 2015	Energy, nutrients, food groups intake vs. family income in urban children			Urban, child care centers and kindergarten	
Philippin es 1	Angeles-Agdeppa 2019	Nutrient and food group intake adequacy in schoolchildren				x
Philippin es 2	Denney 2018	Nutrient and food group intake adequacy in young children				x
Singapore 1	Allan 2017	Validation of FFQ in children 15-36 months	Urban, day care centers			
Singapore 2	Lim 2019	Validation of FFQ in toddlers 18 months	Urban, volunteers			
Singapore 3	Sugianto 2019	Validation of FFQ in 5y old children	Urban, volunteers			
South Korea 1	Baek 2018	Fat and FA intake in children				x
South Korea 2	Kim 2019	N-6 and n-3 PUFA intake vs. recommendations in children				x
Belgium 1	De Ridder 2015	National Survey report				x
Belgium 2	Sioen 2007	N-6 and n-3 PUFA intake in children			Random sampling	
Cyprus	Tornaritis 2014	Nutrient intake vs. body weight and mother education in children and adolescents				x

Country	*Reference*	*Study objective*	*Local* observation study*	*Other type of local* study*	*Sub-national** observation study*	*National survey or nationwide*
Finland 1	Korkalo 2019	Contribution of meals at preschool on total dietary intake			x	
Finland 2	Kyttala 2008	Nutrient intake and food consumption in children			x	
Finland 3	Naveed 2020	FA intake vs. cognition in children		Baseline data of local intervention study (urban, volunteers)		
France 1	Chouraqui 2020	Nutritional intake vs. recommendations in children				x
France 2	Guesnet 2018	PUFA intake in children and adolescents				x
Germany 1	Libuda 2014	Fat intake in children and adolescents (time trend, longitudinal study)	X			
Germany 2	Stahl 2009	Nutrient and food intake in children and adolescents (time trend)				x
Greece	Smpokos 2014	Energy and nutrient intake in children (time trend, 2 cohorts)			Stratified sampling	
Ireland 1	O'Connor 2020	Fat intake in children, time trend				x
Ireland 2	Walton 2017	Nutrient intake vs. recommendations in children				x
Italy	Sette 2011	Nutrient intake, all population				x
Netherlands 1	Goldbohm 2016	Nutrient and food intake in children going to day care centers				x
Netherlands 2	Van Rossum 2020	National Survey Report				x
Poland	Merkiel 2014	Energy and macronutrient intake in children	Preschool members of the Health promotion league			
Spain 1	Lopez-Sobaler 2019	Macronutrient intake vs. recommendations in children and adolescents				x
Spain 2	Madrigal 2020	Fat and FA intake vs. recommendations (vs. family factors) in children			Urban, stratified sampling	
Sweden	Enghardt Barbieri, 2003	National Survey Report				x
Turkey	Rippin 2018	National Survey Report (in a review on children and adolescent nutrient intake)				x

Country	*Reference*	*Study objective*	*Local* observation study*	*Other type of local* study*	*Sub-national** observation study*	*National survey or nationwide*
UK 1	Gibson 2014a	Nutrient intake vs. recommendations in children				x
UK 2	Gibson 2014b	Nutrient intake vs. recommendations in children				x
UK 3	NDNS	National Survey Report				x
UK 4	SACN 2019	Health Agency Report				x
Lebanon	Nasreddine 2018	Nutrient intake vs. status in children (Review using National Surveys)				x
UAE	Ali 2013	Nutrient Intake vs. recommendations in children and adolescents				x
Australia 1	Lioret 2013	Nutrient and food intake in children		Control arm of a RCT, urban, volunteers		
Australia 2	Meyer 2011	N-3 PUFA intake vs. recommendations in children and adolescents				x
Australia 3	Rahmawaty 2013	EPA, DPA, DHA intake and food sources in children and adolescents				x
Australia 4	Rangan 2014	Misreporting of energy intake and adolescents				x
Australia 5	Zhou 2012	Nutrient intake and status in children	Urban, stratified random sampling			
			**N=15**	**N=4**	**N= 8**	**N=38**

Abbreviations: DHA: docosahexaenoic acid; DPA : docosapentaenoic acid (22 :5 n-3) ; EPA: eicosapentaenoic acid; FA: fatty acid; PUFA: polyunsaturated fatty acids; RCT : randomized controlled study

*Sorted by alphabetical order of continent, country and author. **Local: one or several villages or informal settlements, or one town. ***Sub-national: several towns or one county or state or country region.

Table S2 Dietary assessment methods used in the included studies*

Country	*Reference*	*Dietary Recall*	*Dietary Record*	*Weighed or semi-weighed Dietary Records*	*Food Frequency Questionnaire*	*Other method or method combination*
Gambia	Prentice 2000		>1d (#days not specified)			
South Africa 1	Dannhauser 2000	24HR				
South Africa 2	Steyn 2020	24HR + 2x(24HR)				
Canada 1	Barr 2018	24HR				
Canada 2	Health Canada 2012	24HR				

Country	Reference	Dietary Recall	Dietary Record	Weighed or semi-weighed Dietary Records	Food Frequency Questionnaire	Other method or method combination
Canada 3	Innis 2004				FFQ (#days not specified)	
Canada 4	Lien 2009					3d record + FFQ for fish intake
Canada 5	Madden 2009					Other: 3d weighed collection and chemical analysis of all food
Canada 6	Wiedeman 2019		3 d			
Mexico 1	Jimenez-Aguilar 2018				7 d semi-quantitative	
Mexico 2	Ramirez-Silva				7 d	
USA 1	Ahluwalia 2016	2x 24HR				
USA 2	Bailey 2018	24HR + 1x (24HR)				
USA 3	Keim 2015					24HR + 30d FFQ for fish intake
USA 4	Thomson 2019					2x 24HR + 30d FFQ for fish intake
Argentina	Cuesta 2018	24HR				
Brazil	Bueno 2013					1d WR (day care center) + food form (home) + 1x (1d WR + form)
Chile	Rebolledo 2019	24HR + 1x (24HR)				
Bangladesh	Yakes 2011			2d sWR + correction		
China	Barbarich 2006	3x24HR				
Indonesia	Neufingerl 2016	24HR				
Japan 1	Ando 2019				1 month	
Japan 2	Murakami 2018b		3 d + correction			
Japan 3	NHNS-J 2018			1d household WR		
Japan 4	Tsuboyama-Kasaoka 2013			1d household sWR		
Malaysia	Mohd Shariff 2015					24HR + 1x 1d Record
Philippines 1	Angeles-Agdeppa 2019	24HR + 1x (24HR)				
Philippines 2	Denney 2018	24HR + 1x (24HR)				
Singapore 1	Allan 2017			2d WR	3-month (calibrated)	
Singapore 2	Lim 2019	24HR + 1x (24HR)			1-month	
Singapore 3	Sugianto 2019		3d		1-month	

Country	*Reference*	*Dietary Recall*	*Dietary Record*	*Weighed or semi-weighed Dietary Records*	*Food Frequency Questionnaire*	*Other method or method combination*
South Korea 1	Baek 2018	24HR				
South Korea 2	Kim 2019	24HR				
Belgium 1	De Ridder 2015					2 x 24HR + 12-month FFQ
Belgium 2	Sioen 2007		3d			
Cyprus	Tornaritis 2014		3 d			
Finland 1	Korkalo 2019		3 d + 2d record on subsample			
Finland 2	Kyttala 2008		3 d			
Finland 3	Naveed 2020		4 d			
France 1	Chouraqui 2020		3 d			
France 2	Guesnet 2018		7d			
Germany 1	Libuda 2014			3d WR		
Germany 2	Stahl 2009			3 d sWR		
Greece	Smpokos 2014		3 d			
Ireland 1	O'Connor 2020			4d WR + correction		
Ireland 2	Walton 2017			4d WR + correction		
Italy	Sette 2011		3 d			
NL 1	Goldbohm 2016		2 d			
NL 2	Van Rossum 2020					2x24HR combined with food diary + FFQ to assess fish intake
Poland	Merkiel 2014		3 d			
Spain 1	Lopez-Sobaler 2019					2x 1d record + Food Propensity Questionnaire (#days not specified)
Spain 2	Madrigal 2020	2x24HR + correction				
Sweden	Enghardt Barbieri, 2003		4 d			
Turkey	Rippin 2018					24HR (+ FFQ for >5y) (#days not specified)
UK 1	Gibson 2014a		4 d			
UK 2	Gibson 2014b		4 d			
UK 3	NDNS		4 d			

Country	*Reference*	*Dietary Recall*	*Dietary Record*	*Weighed or semi-weighed Dietary Records*	*Food Frequency Questionnaire*	*Other method or method combination*
UK 4	SACN 2019		4d			
Lebanon	Nasreddine 2018	24HR				
UAE	Ali 2013	24HR				
Australia 1	Lioret 2013	2 or 3 x24HR				
Australia 2	Meyer 2014	2x24HR				
Australia 3	Rahmawaty 2013	2x24HR				
Australia 4	Rangan 2014	24HR				
Australia 5	Zhou 2012			3d WR		
Total		**22**<br>– **24HR: 10**<br>– **24HR + x (24HR): 6**<br>– **>1x 24HR: 6**	**19**<br>– **>1d: 18**<br>– **3 d+ (repeat on subsample): 1**	**9**<br>– **1 d: 2**<br>– **>1d: 7**	**7**	**10**

Abbreviations: 24HR: 24-hour dietary recall on whole sample. 24HR + x (24HR): 24 hour dietary recall + x days dietary recall on subsample. x 24HR: x days dietary recall on whole sample. d: day. FFQ: food frequency questionnaire. sWR: semi-weighed record. WR: weighed record.

*Sorted by alphabetical order of continent, country and author.

Table S3 Record of supplements*, fortified foods and breast milk consumption in the included studies**

Country	Reference	*Supplements included*	*Vitamin and mineral supplements included*	*PUFA oil or N-3 oil or fish oil supplements included*	*Fortified foods included*	*Breast milk intake included (children 1-2 y)*
Gambia	Prentice 2000	-	-	-	-	Yes
South Africa 1	Dannhauser 2000	-	-	-	-	>2y
South Africa 2	Steyn 2020	-	-	-	-	Yes
Canada 1	Barr 2018	No	No	No	No	>2y
Canada 2	Health Canada 2012	No	No	No	-	-
Canada 3	Innis 2004	-	-	-	-	-
Canada 4	Lien 2009	-	-	-	-	>2y
Canada 5	Madden 2009	-	-	-	-	>2y
Canada 6	Wiedeman 2019	-	-	No	-	-
Mexico 1	Jimenez-Aguilar 2018	-	-	-	Yes	-
Mexico 2	Ramirez-Silva	-	-	-	-	>2y
USA 1	Ahluwalia 2016	No	No	No	-	Yes
USA 2	Bailey 2018	Yes	Yes	-	-	Yes
USA 3	Keim 2015	-	-	-	-	No
USA 4	Thomson 2019	Yes	Yes	Yes	-	-
Argentina	Cuesta 2018	-	-	-	-	>2y
Brazil	Bueno 2013	-	-	-	-	>2y
Chile	Rebolledo 2019	-	-	-	-	>2y
Bangladesh	Yakes 2011	-	-	-	-	>2y
China	Barbarich 2006	-	-	-	-	-
Indonesia	Neufingerl 2016	-	-	-	-	>2y
Japan 1	Ando 2019	No	No	No	-	>2y
Japan 2	Murakami 2018b	No	No	No	-	>2y
Japan 3	NHNS-J 2018	No	No	No	No	-
Japan 4	Tsuboyama-Kasaoka 2013	No	No	No	No	-
Malaysia	Mohd Shariff 2015	No	No	No	-	No
Philippines 1	Angeles-Agdeppa 2019	No	No	No	-	>2y
Philippines 2	Denney 2018	No	No	No	-	Yes
Singapore 1	Allan 2017	Yes	-	-	Yes	Yes
Singapore 2	Lim 2019	-	-	-	Yes	Yes
Singapore 3	Sugianto 2019	-	-	-	-	>2y

Country	*Reference*	*Supplements included*	*Vitamin and mineral supplements included*	*PUFA oil or N-3 oil or fish oil supplements included*	*Fortified foods included*	*Breast milk intake included (children 1-2 y)*
South Korea 1	Baek 2018	No	No	No	-	No
South Korea 2	Kim 2019	No	No	No	-	No
Belgium 1	De Ridder 2015	Yes	Yes	Yes	Yes	>2y
Belgium 2	Sioen 2007	Yes	Yes	Yes	Yes	>2y
Cyprus	Tornaritis 2014	-	-	-	-	>2y
Finland 1	Korkalo 2019	-	-	-	-	>2y
Finland 2	Kyttala 2008	Yes	Yes	No	-	No
Finland 3	Naveed 2020	-	-	-	-	>2y
France 1	Chouraqui 2020	-	-	-	-	No
France 2	Guesnet 2018	-	-	-	-	>2y
Germany 1	Libuda 2014	-	-	-	-	>2y
Germany 2	Stahl 2009	Yes	Yes	-	Yes	>2y
Greece	Smpokos 2014	-	-	-	-	>2y
Ireland 1	O'Connor 2020	Yes	Yes	Yes	Yes	>2y
Ireland 2	Walton 2017	Yes	Yes	-	Yes	Yes
Italy	Sette 2011	No	No	No	Yes	>2y
NL 1	Goldbohm 2016	Yes	Yes	-	Yes	Yes
NL 2	Van Rossum 2020	Yes	Yes	Yes	Yes	-
Poland	Merkiel 2014	Yes	Yes	-	Yes	>2y
Spain 1	Lopez-Sobaler 2019	No	No	No	Yes	Yes
Spain 2	Madrigal 2020	Yes	Yes	-	Yes	-
Sweden	Enghardt Barbieri, 2003	Yes	Yes	-	-	>2y
Turkey	Rippin 2018	-	-	-	-	
UK 1	Gibson 2014a	Yes	Yes	-	Yes	-
UK 2	Gibson 2014b	Yes	Yes	-	Yes	Yes
UK 3	NDNS	Yes	Yes	-	Yes	-
UK 4	SACN 2019	Yes	Yes	-	Yes	
Lebanon	Nasreddine 2018	-	-	-	-	
UAE	Ali 2013	-	-	-	-	>2y
Australia 1	Lioret 2013	No	No	No	-	Yes
Australia 2	Meyer 2011	Yes	Yes	Yes	Yes	>2y
Australia 3	Rahmawaty 2013	No	No	No	Yes	>2y
Australia 4	Rangan 2014	-	-	-	-	>2y
Australia 5	Zhou 2012	Yes	-	-	-	Yes

Country	*Reference*	*Supplements included*	*Vitamin and mineral supplements included*	*PUFA oil or N-3 oil or fish oil supplements included*	*Fortified foods included*	*Breast milk intake included (children 1-2 y)*
Total Yes		20	18	6	20	13
Total No		16	16	18	3	6
Total -		29	31	41	42	12

Abbreviations: -: not reported or not found

*Supplements= "supplements", "vitamin and mineral supplements" or "n-3 oil" or "fish oil" supplements or "PUFA oil supplements". **Sorted by alphabetical order of continent, country and author.

Table S4 Daily intake of total fat, linoleic, alpha-linolenic acid (%E) and docosahexaenoic acid (mg) in children aged 1 to 2 years (mean or median for boys and girls unless otherwise specified)*

Country	*Reference*	*Total fat*	*LA*	*ALA*	*DHA*
Gambia	Prentice 2000	27.5	5.1	0.23	80
South Africa 2	Steyn 2020	30.3	NR	NR	NR
Canada 2	Health Canada 2012	30.3	NR	0.47	NR
Canada 3	Innis 2004	32	2.98	0.6	41
Canada 6	Wiedeman 2019	NR	3.74	0.53	31
Mexico 1	Jimenez-Aguilar 2018	32.1	NR	NR	NR
USA 1	Ahluwalia 2016	32.9	4.98	0.65	NR
USA 2	Bailey 2018	33	NR	NR	NR
USA 3	Keim 2015	NR	5.5	0.65	19.78
USA 4	Thomson 2019	NR	NR	NR	19.5
China	Barbarich 2006	24.0	2.9	0.4	34
Japan 3	NHNS-J 2018	28.5	4.5	0.57	NR
Japan 4	Tsuboyama-Kasaoka 2013	NR	4.3	0.52	NR
Malaysia	Mohd Shariff 2015[1]	32.61 ; 30.29 ; 32.5	NR	NR	NR
Philippines 2	Denney 2018	29.6	NR	NR	NR
Singapore 1	Allan 2017[2]	29.9; 30.26	NR	NR	100; 120
Singapore 2	Lim 2019[2]	30.2 ; 28.9	NR	NR	NR
South Korea 1	Baek 2018	23.3	3.7	0.5	NR
South Korea 2	Kim 2019	NR	3.5	0.47	37.02
Finland 2	Kyttala 2008	29	NR	NR	NR
France 1	Chouraqui 2020[3]	28.1 ; 29.8	2.8 ; 2.74	0.42 ; 0.38	36; 37

Country	Reference	Total fat	LA	ALA	DHA
Ireland 2	Walton 2017	33.8	NR	0.46	30.3
NL 1	Goldbohm 2016	28.7	5.1	0.7	20
NL 2	Van Rossum 2020[4]	29.5; 29.4	4.6 ; 4.6	0.6 ; 0.6	51 ; 63
Spain 1	Lopez-Sobaler 2019	34.6	NR	NR	NR
Spain 2	Madrigal 2020	36.7	3.7	0.26	80
UK 1	Gibson 2014a	34.1	3.8	0.7	NR
UK 2	Gibson 2014b	35.4	3.7	0.7	NR
UK 3	NDNS	34.4	NR	NR	NR
Australia 1	Lioret 2013	32.75	NR	NR	NR
Australia 5	Zhou 2012	35.5	2.11	0.42	NR

Abbreviations: ALA: alpha-linolenic acid; DHA: docosahexaenoic acid; LA: linoleic acid;

*Studies sorted by alphabetical order of continent, country and author. [1]Children from low income, medium income, and high income families. [2]Dietary assessment using two different methods (Dietary recall or record vs. FFQ). [3]Children aged: 12-17 months; 18-23 months. [4]Mean for: boys; girls.

Table S5 Daily intake of total fat, saturated fats, linoleic acid, alpha-linolenic acid (%E) and sum of docosahexaenoic + eicosapentaenoic acids (mg) in children aged 2 to 3 years (mean or median for boys and girls unless otherwise specified)*

Country	Reference	Total fat	SFA	LA	ALA	EPA+DHA
Gambia	Prentice 2000	15	3.6	4.6	0.13	NR
South Africa 1	Dannhauser 2000[1]	26.4; 26.0	9.6 ; 8.7	5.5 ; 6.5	0.44 ; 0.35	NR
South Africa 2	Steyn 2020	30.3	8.9	NR	NR	NR
Canada 2	Health Canada 2012	30.3	12	NR	0.47	NR
Canada 3	Innis 2004	34.6	13.3	3.5	0.79	152
Canada 6	Wiedeman 2019	NR	NR	4.2	0.59	100
Mexico 1	Jimenez-Aguilar 2018	32.1	14.6	NR	NR	NR
USA 2	Bailey 2018	31	11	NR	NR	NR
USA 3	Keim 2015	NR	NR	5.5	0.59	26.22
USA 4	Thomson 2019	NR	NR	NR	NR	24.7
Brazil	Bueno 2013[2]	28.1 ; 28.5	9.9 ; 9.9	NR	NR	NR
Bangladesh	Yakes 2011	17.2	5.7	3.3	0.4	NR
China	Barbarich 2006	24.0	NR	2.9	0.4	NR
Japan 3	NHNS-J 2018	28.5	9.6	4.5	0.57	NR
Japan 4	Tsuboyama-Kasaoka 2013	NR	NR	4.3	0.52	NR
Malaysia	Mohd Shariff 2015[3]	32.61; 30.29; 32.5	13.37; 12.5; 11.95	NR	NR	NR

Country	Reference	Total fat	SFA	LA	ALA	EPA+DHA
Philippines 2	Denney 2018	23.4	9.4	NR	NR	NR
Singapore 1	Allan 2017[4]	29.9; 30.26	12.09; 12.28	NR	NR	NR
Belgium 2	Sioen 2007	NR	NR	4.03	0.48	65
Finland 2	Kyttala 2008	30	13.2	NR	NR	NR
France 1	Chouraqui 2020[5]	32.6 ; 30.6	NR	2.48 ; 2.17	0.34 ; 0.31	NR
Germany 1	Libuda 2014	34.6	15.2	NR	NR	NR
Ireland 2	Walton 2017	32.8	14.8	NR	0.46	68.2
NL 1	Goldbohm 2016	28.9	10.3	5	0.7	30
NL 2	Van Rossum 2020[6]	29.5; 29.4	11.1; 11.0	4.6;4.6	0.6; 0.6	51; 63
Spain 1	Lopez-Sobaler 2019	34.6	11.9	NR	NR	NR
Spain 2	Madrigal 2020	36.7	13.1	3.7	0.26	130
Turkey	Rippin 2018[6]	37.4 ; 37.0	12.9 ; 12.8	9.1 ; 8.7	0.7 ; 0.7	NR
UK 1	Gibson 2014a	34.1	15.1	3.8	0.7	NR
UK 3	NDNS	34.4	14.5	NR	NR	NR
Lebanon	Nasreddine 2018	38.8	12.7	NR	NR	NR
Australia 2	Meyer 2011	NR	NR	3.3	0.53	NR
Australia 3	Rahmawaty 2013	NR	NR	NR	NR	47.7
Australia 4	Rangan 2014	30.4	13.8	NR	NR	NR
Australia 5	Zhou 2012	34.5	17.2	2.34	0.42	NR

Abbreviations: ALA: alpha-linolenic acid; DHA: docosahexaenoic acid; EPA: eicosapentaenoic acid; LA: linoleic acid; SFA: saturated fatty acids

*Studies sorted by alphabetical order of continent, country and author. [1]Children from: Joe Slovo settlement; JB Mafora settlement. [2]Children from: public schools; private schools. [3]Children from families with: low income; medium income; high income. [4]Dietary assessment using two different methods (Dietary recall or record vs. FFQ). [5]Children aged: 24-29 months; 30-35 months. [6]Mean for: boys; girls.

Table S6 Daily intake of total fat, saturated fats, linoleic acid, alpha-linolenic acid (%E) and docosahexaenoic + eicosapentaenoic acids (mg) in children aged 3 to 5 years (mean or median for boys and girls unless otherwise specified)*

Country	Reference	Total fat	SFA	LA	ALA	EPA+DHA
South Africa 1	Dannhauser 2000	26.4[1];26.0[2]; 25.3[3]; 23.8[4]	9.6[1] ; 8.7[2]; 9.0[3]; 7.9[4]	5.5[1] ; 6.5[2]; 6.2[3]; 5.8[4]	0.44[1];0.35[2] 0.30[3];0.26[4]	NR
South Africa 2	Steyn 2020	28.7	8.5	NR	NR	NR
Canada 2	Health Canada 2012	30.1	12	NR	0.49	NR
Canada 3	Innis 2004	32.4	12	3.7	0.7	156
Canada 5	Madden 2009	22.8	8.4	4.9	0.74	92.5
Mexico 1	Jimenez-Aguilar 2018	32.2	14.2	NR	NR	NR

Country	*Reference*	*Total fat*	*SFA*	*LA*	*ALA*	*EPA+DHA*
USA 2	Bailey 2018	31	11	NR	NR	NR
USA 3	Keim 2015	NR	NR	6.2	0.59	27.21
USA 4	Thomson 2019	NR	NR	NR	NR	24.7
Brazil	Bueno 2013[5]	28.6 ; 28.8	9.8 ; 9.8	NR	NR	NR
Chile	Rebolledo 2019	NR	9.9	NR	NR	NR
Bangladesh	Yakes 2011	12.9	3.4	2.9	0.41	NR
China	Barbarich 2006	21	NR	2.5	0.4	NR
Indonesia	Neufingerl 2016	27.2	11.6	3.3	0.2	30
Japan 1	Ando 2019	NR	NR	4.7	0.5	148.4
Japan 2	Murakami 2018b[6]	29.3 ; 29.2	9.9 ; 9.6	4.3 ; 4.5	0.5 ; 0.5	NR
Japan 3	NHNS-J 2018	28.5	9.6	4.5	0.57	NR
Japan 4	Tsuboyama-Kasaoka 2013	NR	NR	4.5	0.55	NR
Malaysia	Mohd Shariff 2015[7]	32.34 ; 30.79 ; 33.32	11.88; 10.68; 11.65	NR	NR	NR
Philippines 2	Denney 2018	20.2	9.9	NR	NR	NR
South Korea 1	Baek 2018	23	8.3	4	0.6	NR
Belgium 1	De Ridder 2015	32.7	13.5	NR	NR	NR
Belgium 2	Sioen 2007	NR	NR	4.03	0.48	65
Finland 1	Korkalo 2019	31.7	11.7	NR	NR	NR
Finland 2	Kyttala 2008	31	13.2	NR	NR	NR
France 2	Guesnet 2018	38.4	NR	3.7	0.4	184
Germany 1	Libuda 2014	33.8	14.8	NR	NR	NR
Ireland 2	Walton 2017[8]	31.8; 31.5	14.5; 14.1	NR	0.44; 0.44	68; 79.8
Italy	Sette 2011	37.4	11.9	NR	NR	NR
NL 1	Goldbohm 2016	29.3	10.4	5.1	0.7	30
NL 2	Van Rossum 2020[6]	31.7; 31.8	11.8; 11.8	5.0; 5.1	0.6; 0.6	74; 81
Spain 1	Lopez-Sobaler 2019[6]	34.8; 35.6	13.1; 13.1	NR	NR	NR
Spain 2	Madrigal 2020	36.8	13.5	3.8	0.27	130
Sweden	Enghardt Barbieri 2003	31.7	14.4	3	0.6	140
Turkey	Rippin 2018[6]	37.4 ; 37.0	12.9 ; 12.8	9.1 ; 8.7	0.7 ; 0.7	NR
UK 3	NDNS	33.4	13	NR	NR	NR
UK 4	SACN 2019	NR	NR	4.4	0.8	NR
Lebanon	Nasreddine 2018	38.8	12.7	NR	NR	NR
Australia 2	Meyer 2011	NR	NR	3.5	0.57	NR
Australia 3	Rahmawaty 2013	NR	NR	NR	NR	55.1
Australia 4	Rangan 2014	30.4	13.8	NR	NR	NR
Australia 5	Zhou 2012	32.1	14.9	2.6	0.34	NR

Abbreviations: ALA: alpha-linolenic acid; DHA: docosahexaenoic acid; EPA: eicosapentaenoic acid; LA: linoleic acid; SFA: saturated fatty acids

*Studies sorted by alphabetical order of continent, country and author. [1]Children 2 to 3.9 years from Joe Slovo settlement. [2]Children 2 to 3.9 years from JB Mafora settlement. [3]Children 4 to 5.9 years from Joe Slovo settlement. [4]Children 4 to 5.9 years from JB Mafora settlement. [5]Children from: public schools; private schools. [6]Mean for: boys; girls. [7]Children from families with: low income; medium income; high income. [8]Children aged: 3 years; 4 years.

Table S7 Daily intake of total fat, saturated fats, linoleic acid, alpha-linolenic acid (%E) and docosahexaenoic + eicosapentaenoic acids (mg) in children aged 5 to 7 years (mean or median for boys and girls unless otherwise specified)*

Country	*Reference*	*Total fat*	*SFA*	*LA*	*ALA*	*DHA*
South Africa 1	Dannhauser 2000[1]	25.3;23.8	9.0 ; 7.9	6.2 ; 5.8	0.30 ; 0.26	NR
South Africa 2	Steyn 2020	28.7	8.5	NR	NR	NR
Canada 1	Barr 2018	30.1	10.9	NR	NR	NR
Canada 2	Health Canada 2012	30.1	12	NR	0.49	NR
Canada 4	Lien 2009	33	12	3.9	0.36	54
Canada 5	Madden 2009	22.8	8.4	4.9	0.74	92.5
Mexico 2	Ramirez-Silva 2011	26.7	11.4	3.2	0.2	NR
USA 4	Thomson 2019	NR	NR	NR	NR	50.8
Argentina	Cuesta 2018[2]	36.7 ; 37.6	11.4 ; 13.1	NR	NR	NR
Brazil	Bueno 2013[3]	28.6 ; 28.8	9.8 ; 9.8	NR	NR	NR
Indonesia	Neufingerl 2016	27.2	11.6	3.3	0.2	30
Japan 2	Murakami 2018b[2]	29.3 ; 29.2	9.9 ; 9.6	4.3 ; 4.5	0.5 ; 0.5	NR
Japan 3	NHNS-J 2018	28.5	9.6	4.5	0.57	NR
Japan 4	Tsuboyama-Kasaoka 2013	NR	NR	4.5	0.67	NR
Malaysia	Mohd Shariff 2015[4]	32.34 ; 30.79 ; 33.32	11.88; 10.66; 11.65	NR	NR	NR
Philippines 1	Angeles-Agdeppa 2019	17.8	9.27	NR	NR	NR
Singapore 3	Sugianto 2019[5]	26.5 ; 25.0	9.1 ; 9.3	NR	NR	NR
Belgium 1	De Ridder 2015	34.6	13.2	NR	NR	NR
Belgium 2	Sioen 2007	NR	NR	4.25	0.54	75
Cyprus	Tornaritis 2014[2]	34.7 ; 34.1	14 ; 13.7	NR	NR	NR
Finland 1	Korkalo 2019	31.9	11.5	NR	NR	NR
Finland 2	Kyttala 2008	31	13.7	NR	NR	NR
Finland 3	Naveed 2020	30	12.2	3.6	0.78	140
France 2	Guesnet 2018	38.2	NR	3.5	0.4	144
Germany 1	Libuda 2014	33.8	14.8	NR	NR	NR

Country	*Reference*	*Total fat*	*SFA*	*LA*	*ALA*	*DHA*
Germany 2	Stahl 2009	32	14	NR	NR	NR
Greece	Smpokos 2014[2]	42.8 ; 43.1	15.4 ; 15.3	3.5 ; 3.5	0.38 ; 0.28	NR
Ireland 1	O'Connor 2020	33.1	14.2	3.5	0.6	100.3
Italy	Sette 2011	37.4	11.9	NR	NR	NR
NL 2	Van Rossum 2020[2]	31.7; 31.8	11.8; 11.8	5.0; 5.1	0.6; 0.6	74; 81
Poland	Merkiel 2014	32.7	14.5	NR	NR	NR
Spain 1	Lopez-Sobaler 2019[2]	34.8; 35.6	13.1; 13.1	NR	NR	NR
Spain 2	Madrigal 2020	36.8	13.5	3.8	0.27	130
Turkey	Rippin 2018[3]	35.2 ; 35.8	12.0 ; 11.7	8.8 ; 9.3	0.6 ; 0.7	NR
UK 3	NDNS	33.4	13	NR	NR	NR
UK 4	SACN 2019	NR	NR	4.4	0.8	NR
UAE	Ali 2013[3]	26.3; 24.4	9.77; 8.9	NR	NR	NR
Australia 2	Meyer 2011	NR	NR	3.5	0.57	NR
Australia 3	Rahmawaty 2013	NR	NR	NR	NR	55.1
Australia 4	Rangan 2014	30.4	13.8	NR	NR	NR

Abbreviations: ALA: alpha-linolenic acid; DHA: docosahexaenoic acid; EPA: eicosapentaenoic acid; LA: linoleic acid; SFA: saturated fatty acids

*Studies sorted by alphabetical order of continent, country and author. [1]Children from: Joe Slovo settlement; JB Mafora settlement. [2]Mean for: boys; girls. [3]Children from: public schools; private schools. [4]Children from families with: low income; medium income; high income. [5]Dietary assessment using two different methods (Dietary recall or record vs. FFQ)
